# Assessment of the dietary amino acid profiles and the relative biomarkers for amino acid balance in the low-protein diets for broiler chickens

**DOI:** 10.1186/s40104-024-01108-2

**Published:** 2024-11-14

**Authors:** Bin Wang, Xiaodan Zhang, Yongfa Liu, Mingkun Gao, Mi Wang, Yuan Wang, Xinzhi Wang, Yuming Guo

**Affiliations:** 1 State Key Laboratory of Animal Nutrition and Feeding, College of Animal Science and Technology, China Agricultural University, Beijing, 100193 China; 2 Shenyang Boeing Feed Company, Shenyang, 110141 China

**Keywords:** Amino acid balance, Biomarkers, Essential amino acid, Low-protein diet, Metabolomics

## Abstract

**Background:**

Research on low-protein-level diets has indicated that even though the profiles of essential amino acids (EAAs) follow the recommendation for a normal-protein-level diet, broilers fed low-protein diets failed to achieve productive performance compared to those fed normal diets. Therefore, it is imperative to reassess the optimum profile of EAAs in low-protein diets and establish a new ideal pattern for amino acid balance. Furthermore, identifying novel sensitive biomarkers for assessing amino acid balance will greatly facilitate the development of amino acid nutrition and application technology. In this study, 12 dietary treatments [Con(+), Con(-), L&A(-), L&A(+), M&C(-), M&C(+), BCAA (-), BCAA(+), Thr(-), Thr(+), Trp(-) and Trp(+)] were established by combining different EAAs including lysine and arginine, methionine and cysteine, branched-chain amino acid (BCAA), threonine, and tryptophan to observe the growth and development of the broiler chickens fed with low-protein-level diets. Based on the biochemical parameters and untargeted metabolomic analysis of animals subjected to different treatments, biomarkers associated with optimal and suboptimal amino acid balance were identified.

**Results:**

Growth performance, carcass characteristics, hepatic enzyme activity, serum biochemical parameters, and breast muscle mRNA expression differed significantly between male and female broilers under different dietary amino acid patterns. Male broilers exhibited higher sensitivity to the adjustment of amino acid patterns than female broilers. For the low-protein diet, the dietary concentrations of lysine, arginine, and tryptophan, but not of methionine, cystine, or threonine, needed to be increased. Therefore, further research on individual BCAA is required. For untargeted metabolomic analysis, Con(+) was selected as a normal diet (NP) while Con(-) represented a low-protein diet (LP). L&A(+) denotes a low-protein amino acid balanced diet (LPAB) and Thr(+) represents a low-protein amino acid imbalance diet (LPAI). The metabolites oxypurinol, pantothenic acid, and D-octopine in birds were significantly influenced by different dietary amino acid patterns.

**Conclusion:**

Adjusting the amino acid profile of low-protein diets is required to achieve normal growth performance in broiler chickens fed normal-protein diets. Oxypurinol, pantothenic acid, and D-octopine have been identified as potentially sensitive biomarkers for assessing amino acid balance.

**Supplementary Information:**

The online version contains supplementary material available at 10.1186/s40104-024-01108-2.

## Introduction

Low-protein diets have received significant attention in the broiler industry because of their significant role in reducing nitrogen emissions [1–3]. Low-protein diets were formulated to meet amino acid nutritional requirements by incorporating crystalline amino acids [4]; however, long-term feeding of low-protein diets in broilers results in an increase in fat pad rate (FR) and feed conversion ratio (FCR) [5–7], nevertheless. Several studies have indicated that broilers fed with varying patterns of amino acids exhibit notable enhancements in body weight (BW) and FCR, as well as significant alterations in carcass characteristics [8–10]. Furthermore, Macelline et al. [11] conducted a study on broilers fed diets with two different protein contents and amino acid patterns and revealed that the optimal growth of broilers necessitates distinct amino acid patterns depending on the protein content. Consequently, it proved challenging to meet bird growth requirements by formulating a low-protein diet based solely on the amino acid patterns established in a normal-protein diet.

During protein synthesis, animals require the combined participation of essential amino acids (EAAs) and non-essential amino acids (NEAAs). Numerous studies have demonstrated that animals fed low-protein diets exhibit poor growth performance, accompanied by a reduction in blood NEAA concentration [12–14], indicating that NEAAs or total nitrogen deficiency may limit animal growth [15]. As the total protein content decreases in a low-protein diet, the body’s supply of nitrogen used for synthesizing NEAAs also decreases, resulting in the conversion of some dietary EAAs into NEAAs through oxidative decomposition. Therefore, it is imperative to reassess EAA concentration in low-protein diets to determine the optimal EAA pattern.

In current broiler feeding experiments, the main methods for evaluating dietary amino acids are addition and deletion assays, each with its advantages and disadvantages [16]. Combining these two methods effectively allows for the determination of increasing and decreasing trends in amino acid concentrations in the diet by comparing differences in animal traits when amino acids are present or absent. Furthermore, it is important to note the interactions among amino acids of the same type; therefore, any change in the concentration of one amino acid will have a certain impact on another [17, 18]. Therefore, EAAs can be categorized into different combinations based on their structural and functional properties. The pattern of each amino acid combination in the diet was adjusted using a combination of addition and deletion methods to evaluate the changes in amino acid composition.

The indices commonly used for assessing dietary amino acid balance include the nitrogen balance assay and serum urea nitrogen or uric acid concentration [19]. These indices are based on the excretion or deposition of total nitrogen in the diet. However, the nitrogen balance assay is a cumbersome procedure with significant variation, hindering its large-scale application in production. As mentioned earlier, dietary total nitrogen is involved in NEAA synthesis, indicating that serum urea nitrogen or uric acid concentrations do not fully reflect the extent of amino acid balance. Therefore, it is necessary to identify biomarkers that accurately reflect amino acid balance. Metabolomics serves as a systematic biological analysis tool for studying amino acid nutrition [20]; it intuitively reflects metabolic differences among animals under different conditions and assesses how metabolic balance is affected by amino acid deficiency or excess [21, 22]. Subsequently, diets with optimal and suboptimal amino acid balances were selected, followed by untargeted metabolomic analysis to identify biomarkers for assessing amino acid balance.

## Materials and methods

### Animal ethics

This study was conducted at the Zhuozhou Experimental Base of China Agricultural University and approved by the Animal Welfare and Ethics Review Committee of China Agricultural University (AW61504202-1-2).

### Experimental diets

The feeding standard for the experimental diet was based on the Feeding Standard of Chickens (2004) [23], with equal energy and electrolyte balance. Starter, grower, and finisher phases had a protein content of 23% (0–14 d), 21% (15–28 d), and 20% (29–42 d), respectively, in the positive control normal diet. Further, the negative control had a protein content of 21%, 19%, and 18% in the starter, grower, and finisher phases, respectively, and the low-protein diet, dietary composition, and nutrient concentration are shown in Table 1. Dietary EAAs were divided into five combinations according to their structure and properties: lysine and arginine, methionine, cysteine, branched-chain amino acids (BCAA), threonine, and tryptophan. According to the principles of addition and deletion methods, the five amino acid combinations were adjusted by 10% based on a low-protein diet (Table 2). The specific treatments were: the normal-protein control diet [Con(+)], the low-protein control diet [Con(-)], the lysine and arginine restriction diet [L&A(-)], the lysine and arginine supplementation diet [L&A(+)], the methionine and cysteine restriction diet [M&C(-)], the methionine and cysteine supplementation diet [M&C(+)], the BCAA restriction diet [BCAA(-)], the BCAA supplementation diet [BCAA(+)], the threonine restriction diet [Thr(-)], the threonine supplementation diet [Thr(+)], the tryptophan restriction diet [Trp(-)], and the tryptophan supplementation diet [Trp(+)], detailed dietary composition and nutrient concentration are shown in Additional file 1: Table S1–S3. All feeds were mixed evenly in a vertical spiral mixer (DLH-30, Guibao) and granulated using steam at 80 °C.

**Table 1:** Ingredients and nutrient composition of the experimental diets (as-fed basis^1^)

Item	Starter phase (0–14 d)		Grower phase (15–28 d)		Finisher phase (29– 42 d)	
	NP^2^	LP	NP	LP	NP	LP
Corn (level 2, 7.8%), %	53.51	57.22	57.75	66.34	62.02	65.26
Soybean meal (level 3, 44%), %	29.94	28.37	29.43	19.67	22.07	21.15
Corn gluten meal (61%), %	7.99	4.18	5.07	4.51	8.11	2.75
Calcium bicarbonate (anhydrous), %	1.53	1.69	1.43	1.50	1.21	1.28
Soy oil, %	4.18	4.88	3.97	3.30	4.24	5.44
Mineral meal, %	1.39	1.33	1.21	1.24	1.20	1.16
Salt, %	0.33	0.31	0.31	0.24	0.28	0.27
Choline chloride (50%), %	0.23	0.23	0.22	0.26	0.20	0.21
Mineral premix^3^, %	0.20	0.20	0.20	0.20	0.20	0.20
DL-Methionine, %	0.13	0.18	0.13	0.17	0.06	0.14
L-Lysine HCl, %	0.18	0.23	0.11	0.33	0.18	0.24
Vitamin premix^4^, %	0.02	0.02	0.02	0.02	0.02	0.02
L-Cysteine, %	0.01	0.03	0.02	0.06	0.01	0.04
L-Phenylalanine, %	0.00	0.04	0.01	0.21	0.00	0.33
L-Threonine, %	0.00	0.04	0.00	0.13	0.00	0.10
L-Arginine, %	0.13	0.21	0.01	0.29	0.01	0.11
L-Histidine, %	0.00	0.00	0.01	0.10	0.01	0.07
L-Isoleucine, %	0.00	0.00	0.00	0.16	0.00	0.11
L-Leucine, %	0.00	0.31	0.00	0.27	0.00	0.51
L-Tryptophan, %	0.06	0.03	0.00	0.05	0.01	0.01
L-Valine, %	0.00	0.02	0.00	0.17	0.01	0.14
Glycine, %	0.00	0.19	0.00	0.30	0.00	0.24
Phytase, %	0.02	0.02	0.02	0.02	0.02	0.02
Potassium carbonate, %	0.14	0.19	0.01	0.26	0.01	0.05
Sodium bicarbonate, %	0.03	0.06	0.09	0.20	0.13	0.16
Nutrient concentration						
Analyzed^5^						
SID CP%	23.21	21.08	21.15	19.12	20.22	18.21
SID Lysine, %	1.21	1.17	1.11	1.06	0.98	0.98
SID Methionine, %	0.50	0.49	0.47	0.48	0.41	0.40
SID Cysteine, %	0.41	0.39	0.37	0.35	0.36	0.35
SID Methionine + Cysteine, %	0.91	0.87	0.84	0.83	0.77	0.75
SID Phenylalanine + Tyrosine, %	1.64	1.51	1.35	1.27	1.45	1.33
SID Phenylalanine, %	0.97	0.96	0.79	0.81	0.93	0.88
SID Tyrosine, %	0.67	0.56	0.56	0.47	0.52	0.46
SID Threonine, %	0.83	0.79	0.73	0.72	0.68	0.67
SID Arginine, %	1.61	1.44	1.28	1.23	1.10	1.19
SID Histidine, %	0.55	0.53	0.54	0.43	0.46	0.42
SID Isoleucine, %	0.93	0.81	0.87	0.76	0.72	0.73
SID Leucine, %	2.15	2.05	2.06	2.03	2.14	2.03
SID Tryptophan, %	0.29	0.24	0.21	0.19	0.17	0.17
SID Valine, %	1.05	1.02	0.94	0.95	0.91	0.90
SID Glycine, %	1.72	1.89	2.06	1.50	2.03	1.36
SID Glycine + Serine, %	0.74	0.96	1.23	0.77	1.20	0.60
SID Serine, %	0.98	0.93	0.83	0.72	0.83	0.76
Calculated^6^						
Dry matter, %	89.30	89.23	89.14	89.08	89.32	89.16
ME, Mcal/kg	3.13	3.07	3.12	3.14	3.21	3.20
Calcium, %	1.05	1.05	0.89	0.99	0.91	0.91
Available phosphorus, %	0.34	0.34	0.29	0.29	0.30	0.30
Kalium, %	0.78	0.78	0.70	0.70	0.59	0.59
Natrium, %	0.16	0.16	0.16	0.16	0.16	0.16
Chlorine, %	0.28	0.28	0.25	0.25	0.25	0.25
Choline, mg/kg	2344.19	2344.24	2299.51	2299.47	2046.47	2047.22
Electrolyte balance, mmol/kg	189.49	189.48	178.46	178.43	152.59	152.57

^1^Ingredients and nutrient composition of the experimental diets were based on air dry matter

^2^NP: Normal CP diet; LP: Low-CP diet

^3^The Mineral premix provided the following per kg of diet: Cu: 16 mg; Zn: 110 mg; Fe: 80 mg; Mn: 120 mg; Se: 0.30 mg; I: 1.50 mg

^4^The Vitamin premix provided the following per kg of diet: Vitamin A: 15,000 IU; Vitamin D_3_: 3600 IU; Vitamin E: 30 IU; Vitamin K_3_: 3.00 mg; Vitamin B_2_: 9.60 mg; Vitamin B_12_: 0.03 mg; Biotin: 0.15 mg; Folic acid: 1.50 mg; Pantothenic acid: 13.80 mg; Niacin: 45 mg

^5^The following data are analyzed, the relevant analytical methods have been indicated in the Material and method

^6^The following data are calculated according to the Feeding Standard of Chickens (2004) [23]

**Table 2:** The ratio of different amino acid combinations of the experimental diets

Item	CP^1^	L&A^2^	M&C^2^	BCAA^2^	Thr^2^	Trp^2^
Con(+)	23%, 21%, 20%	100%	100%	100%	100%	100%
Con(-)	21%, 19%, 18%	100%	100%	100%	100%	100%
L&A(-)	21%, 19%, 18%	90%	100%	100%	100%	100%
L&A(+)	21%, 19%, 18%	110%	100%	100%	100%	100%
M&C(-)	21%, 19%, 18%	100%	90%	100%	100%	100%
M&C(+)	21%, 19%, 18%	100%	110%	100%	100%	100%
BCAA(-)	21%, 19%, 18%	100%	100%	90%	100%	100%
BCAA(+)	21%, 19%, 18%	100%	100%	110%	100%	100%
Thr(-)	21%, 19%, 18%	100%	100%	100%	90%	100%
Thr(+)	21%, 19%, 18%	100%	100%	100%	110%	100%
Trp(-)	21%, 19%, 18%	100%	100%	100%	100%	90%
Trp(+)	21%, 19%, 18%	100%	100%	100%	100%	110%

Con(+): The normal-protein control group; Con(-): The low-protein control group; L&A(-): The lysine and arginine restriction group; L&A(+): The lysine and arginine supplementation group; M&C(-): The methionine and cysteine restriction group; M&C(+): The methionine and cysteine supplementation group; BCAA(-): The BCAA restriction group; BCAA(+): The BCAA supplementation group; Thr(-): The threonine restriction group; Thr(+): The threonine supplementation group; Trp(-): The tryptophan restriction group; Trp(+): The tryptophan supplementation group

^1^The three values of CP indicate the protein content in the diet at the starter, grower and finisher phases, respectively

^2^The percentage of each amino acid represents the ratio of the corresponding amino acid in the diet to Con(+) or Con(-)

All the samples of experimental diets were polished into powder and stored at −20 °C to determine the content of crude protein, energy, and EAA concentrations.

### Animals and feeding management

A total of 720 1-day-old healthy AA broilers, half male, and half female (identified by feather speed) with an initial BW of 43.5 ± 0.5 g, were randomly assigned to 12 diets according to sex with 6 pens per treatment and 10 broilers per pen. The experiment lasted for 40 d and was divided into 3 phases: the starter phase (0–14 d), grower phase (15–28 d), and finisher phase (29–40 d). The broilers were kept captive, with free access to feed and water. The illumination period of the poultry houses was gradually reduced from 24 h/d to 20 h/d. The temperature was gradually lowered from 35 to 22 °C. The immunization procedure was carried out according to the management manual, and the environment of the poultry house and health status of the broilers were checked daily.

### Sample collection and analysis

All animal experiments were performed using the same procedures. The BW of each pen was measured, feed consumption was recorded at the beginning and end of each phase, and the number of deaths and the BW of dead broilers were recorded daily to correct the growth performance data. At 40 d, 4 broilers close to the average BW were selected from each pen, and 2 were used for slaughter. The slaughtering process was in accordance with the Operating Procedure of Livestock and Poultry Slaughtering—Chicken [24], and the measurements included dressing, half-eviscerated, eviscerated, fat pad, breast, and thigh rates. The remaining 2 were used for sample collection. After the broilers were euthanized by electric shock, blood was quickly collected from the jugular vein and placed in a coagulant tube. After standing at room temperature for 4 h, the blood was transferred to a 4 °C centrifuge, centrifuged at 3,000 r/min for 15 min, and the supernatant was stored at −80 °C. Liver and chest muscle tissues were collected and placed in a sterile cryopreservation tube, and after quenching with liquid nitrogen, they were transferred to −80 °C for preservation.

### Serum biochemical parameter analysis

Serum concentrations of total protein (TP), albumin (ALB), globulin (GLB), triglyceride (TG), total cholesterol (TC), blood urea nitrogen (BUN), uric acid (UA), glucose (GLU), alanine transaminase (ALT), and aspartate aminotransferase (AST), were analyzed using an automatic biochemical analyzer (BK1200, Biobase, Jinan, Shandong, China). All the operating procedures were performed according to the manufacturer’s instructions.

### Hepatic enzyme activities analysis

The liver tissue was mixed and ground with normal saline, and the supernatant was removed after centrifugation and transferred to 4 °C for storage. Aspartate aminotransferase (AST), alanine aminotransferase (ALT), acetyl-CoA carboxylase (ACC), fatty acid synthase (FAS), glucose-6-phosphatase (G6PC), pyruvate carboxylase (PC), HMG-CoA reductase (HMGR), and xanthine oxidase (XOD) were measured using commercial reagent kits (Nanjing Jiancheng Bioengineering Institute, Nanjing, China). All procedures were performed according to the manufacturer’s instructions.

### Real-time quantitative PCR

RNA extraction, reverse transcription, and qRT-PCR were performed as described by Yang et al. [25]. Total RNA was extracted from the breast muscle tissue using RNAiso Plus reagent (Takara, Beijing, China). After the purity and concentration of RNA were determined using a nucleic acid analyzer, cDNA was synthesized using a reverse transcription kit, diluted, and analyzed using qRT-PCR. The resulting mRNA expression was normalized to *GAPDH*, and the remaining relative mRNA expression was calculated. Primers for *GAPDH*, *4EBP1*, *S6K1*, *MAFbx*, and *MuRF1* were designed and commercially synthesized according to sequences in GenBank (Table 3) (Sangon Biotechnology, Shanghai, China).

**Table 3:** Parameters of primer pairs for qRT-PCR

Genes^1^	GenBank number	Sequence (5′→3′)
*GAPDH*	NM_204305.1	F: GAGGGTAGTGAAGGCTGCTG
		R: CATCAAAGGTGGAGGAATGG
*S6K1*	NM_001030721.1	F: TGGACCATGGAGGAGTTGG
		R: AGCACTCCGGTCGGATCTT
*4EBP1*	XM_424384.5	F: GTTCCTGATGGAGTGCCGTA
		R: GTTCCTGATGGAGTGCCGTA
*MAFbx*	NM_001030956.1	F: CCTTCACAGACCTGCCATTG
		R: GCAGAGCTTCTTCCACAGCA
*MuRF1*	XM_015297755.1	F: GCTGGTGGAGAACATCATCG
		R: TCGCAGGTGACGCAGTAGAT

^1^*GAPDH *Glyceraldehyde-3-phosphate dehydrogenase, *S6K1 *The 70-kDa ribosomal protein S6 kinase, *4EBP1 *Eukaryotic translation initiation factor 4E-binding protein-1, *MAFbx *Muscle atrophy f-box, *MuRF1 *Muscle ring finger 1

### Metabolite extraction and detection

At low temperature, 25 mg sample was weighed into an EP tube, and 500 µL extraction solution (methanol:acetonitrile:water=2:2:1; v/v) containing isotope labeled internal standard mixture was added. The mixture was vortexed and stirred for 30 s. The homogenate beads were added and homogenized in a homogenizer (35 Hz, 4 min), transferred to an ice water bath, sonicated for 5 min, and the process was repeated 3 times. The homogenate was left at −40 °C for 1 h before being transferred to a centrifuge, and centrifuged at 12,000 r/min for 15 min at 4 °C. The supernatant was collected for further examination.

Chromatographic separation of the target compounds was performed using an ultra-high performance liquid chromatograph with a chromatographic column (Waters ACQUITY UPLC BEH Amide; 2.1 mm × 50 mm, 1.7 μm). Liquid chromatography phase A was aqueous (25 mmol/L ammonium acetate and 25 mmol/L aqueous ammonia), and phase B was acetonitrile. Sample plate temperature: 4 °C. The injection volume was 2 µL. Primary and secondary mass spectrometry data were acquired using Orbitrap Exploris 120 mass spectrometer (Xcalibur, version: 4.4, Thermo).

### Data preprocessing and metabolite identification

The raw metabolome data were converted into mzXML format by ProteoWizard software, and then metabolites were visualized by R language.

The identification method of metabolites is as described by Marín-García et al. [26]. Local self-built data databases and public databases were used to search databases [27]. By matching the retention time, molecular mass (molecular mass error within < 10 ppm), secondary fragmentation spectrum, collision energy and other information of metabolites in the database, the structure of metabolites in biological samples was identified, and the identification results were strictly manually checked and confirmed. The identification Level was Level 2 and above.

### Statistical analysis

Each pen was considered as an experimental unit. One-way ANOVA was used for data analysis using SPSS 27 (IBM), and the results are presented as the means. *P* < 0.05 was considered statistically significant difference, 0.05 < *P* < 0.1 indicated a trend of difference. To clarify the adjustment trends of different EAA combinations, growth performance and carcass characteristics were analyzed for the same amino acid combinations. Hepatic enzyme activity and serum biochemical parameters were graphically plotted using Origin 2022 software (OriginLab).

Principal component analysis (PCA) was used to outline the population structure, and orthogonal partial least squares discriminant analysis (OPLS-DA) was used to analyze differences between treatments. The goodness of fit (*R*^2^) and goodness of prediction (*Q*^2^) were used to represent the cross-validation results, and the closer they were to 1, the more reliable the model was. The *t*-test and volcano plot were used for data analysis, and metabolites with VIP > 1, fold change > 2 or < 0.5 and *P* < 0.05 were considered differential metabolites. A Venn plot was used to show the shared differential metabolites among the four treatments, and their potential as markers was determined based on the receiver operating characteristic (ROC) curve, with an area under the curve (AUC) closer to 1 indicating greater potential. In addition, the Kyoto Encyclopedia of Genes and Genomes (KEGG) and *Gallus gallus* (chicken) metabolome database [28] were used to enrich metabolic pathways related to differential metabolites, with higher percentages of metabolites and rich factors indicating more significant enrichment of the pathway.

## Results

### Growth performance

#### Lysine and arginine allowance

In male broilers, significant differences across the four treatment groups were observed in BW, average daily feed intake (ADFI), average daily gain (ADG), and FCR throughout the various growth phases. The lysine- and arginine-supplemented groups demonstrated the best overall performance. Specifically, this group had the highest BW during the grower and finisher phases, albeit slightly lower than that of the low-protein control group during the starter phase; ADFI was lower than that of the low-protein control group in the early phases but peaked in the finisher phase. The Lysine and Arginine supplemented groups consistently had the highest ADG, except for the starter phase. Conversely, the normal protein control group exhibited the lowest FCR except during the finishing phase.

For female broilers, the patterns of significant differences in BW, ADFI, ADG, and FCR among the treatments were similar, although the differences in the grower phase were less pronounced than those in males, yet discernible trends were present. Throughout all phases, the Lysine and Arginine supplemented groups outperformed the other treatment groups in terms of BW, ADFI, and ADG. The normal-protein control group consistently had the lowest FCR, except in the finisher phase (Table 4).

**Table 4:** Effects of different lysine and arginine allowance on growth performance of AA broilers

Item		**Con(+)**	**Con(-)**	**L&A(-)**	**L&A(+)**	**SEM**	***P*-value**
Male							
0–14 d	IBW	0.044	0.043	0.044	0.043	< 0.001	0.423
	FBW	0.347^b^	0.383^a^	0.341^b^	0.371^a^	0.004	< 0.001
	ADFI	0.029^b^	0.034^a^	0.029^b^	0.032^a^	0.001	0.007
	ADG	0.022^bc^	0.025^a^	0.021^c^	0.023^bc^	< 0.001	0.005
	FCR	1.304	1.363	1.377	1.402	0.027	0.182
15–28 d	FBW	1.244^b^	1.262^b^	1.262^b^	1.296^a^	0.008	0.020
	ADFI	0.094^b^	0.104^a^	0.101^a^	0.101^a^	0.002	0.014
	ADG	0.064^ab^	0.063^b^	0.065^a^	0.066^a^	0.001	0.054
	FCR	1.462^b^	1.656^a^	1.545^ab^	1.533^b^	0.032	0.025
29–40 d	FBW	2.338^bc^	2.313^c^	2.375^b^	2.508^a^	0.016	< 0.001
	ADFI	0.145^b^	0.148^b^	0.154^a^	0.159^a^	0.002	0.004
	ADG	0.091^b^	0.087^b^	0.093^b^	0.101^a^	0.002	0.012
	FCR	1.591	1.690	1.667	1.576	0.038	0.227
0–40 d	ADFI	0.086^c^	0.092^b^	0.092^b^	0.094^a^	0.001	< 0.001
	ADG	0.058^b^	0.057^b^	0.058^ab^	0.062^a^	0.001	0.063
	FCR	1.499^b^	1.631^a^	1.576^ab^	1.532^b^	0.026	0.039
Female							
0–14 d	IBW	0.043	0.044	0.044	0.044	< 0.001	0.680
	FBW	0.340^b^	0.361^ab^	0.335^b^	0.387^a^	0.009	0.024
	ADFI	0.029^bc^	0.032^ab^	0.029^c^	0.034^a^	0.001	0.007
	ADG	0.021^b^	0.023^ab^	0.021^b^	0.024^a^	0.001	0.044
	FCR	1.362	1.370	1.367	1.384	0.022	0.946
15–28 d	FBW	1.195^b^	1.194^b^	1.243^ab^	1.311^a^	0.027	0.090
	ADFI	0.090^b^	0.094^ab^	0.097^ab^	0.101^a^	0.002	0.036
	ADG	0.061^ab^	0.059^b^	0.065^a^	0.066^a^	0.002	0.076
	FCR	1.480^b^	1.588^a^	1.495^b^	1.543^ab^	0.024	0.060
29–40 d	FBW	2.235^b^	2.194^b^	2.243^b^	2.422^a^	0.035	0.020
	ADFI	0.137	0.140	0.141	0.152	0.004	0.169
	ADG	0.087^b^	0.083^b^	0.083^b^	0.093^a^	0.002	0.016
	FCR	1.588	1.685	1.701	1.643	0.039	0.441
0–40 d	ADFI	0.083^b^	0.086^b^	0.086^ab^	0.093^a^	0.002	0.042
	ADG	0.055^b^	0.054^b^	0.055^b^	0.059^a^	0.001	0.002
	FCR	1.517	1.605	1.571	1.565	0.026	0.231

*Con(+) *The normal-protein control group, *Con(-)* The low-protein control group, *L&A(-)* The lysine and arginine restriction group, *L&A(+) *The lysine and arginine supplementation group, *IBW* Initial body weight, *FBW* Final body weight, *ADFI* Average daily feed intake, *ADG* Average daily gain, *FCR* Feed:Gain, *SEM* Standard error of the mean

^a−c^Dissimilar letters represent significant difference among different treatments (*P* < 0.05)

#### Methionine and cysteine allowance

During the starter phase, significant differences were noted in ADFI among the four treatments in male broilers. Similarly, in the grower phase, a notable divergence was observed in the FCR. Trends in the BW and ADG were discernible during the starter phase. However, no significant variations were detected in the remaining indicators. The normal protein control group had the lowest BW, ADFI, and ADG in the starter phase, and the lowest FCR in the grower phase. Female broilers showed the same performance as male broilers, but the methionine and cysteine restriction groups had the lowest FCR in the starter phase and the highest ADFI in the finisher phase (Table 5).

**Table 5:** Effects of different methionine and cysteine allowance on growth performance of AA broilers

**Item**		**Con(+)**	**Con(-)**	**M&C(-)**	**M&C(+)**	**SEM**	***P*-value**
Male							
0–14 d	IBW	0.044	0.043	0.044	0.043	< 0.001	0.355
	FBW	0.347^b^	0.383^ab^	0.392^a^	0.391^a^	0.010	0.083
	ADFI	0.029^b^	0.034^a^	0.034^a^	0.033^a^	0.001	0.016
	ADG	0.022^b^	0.025^a^	0.025^a^	0.025^a^	0.001	0.052
	FCR	1.304	1.363	1.349	1.329	0.033	0.705
15–28 d	FBW	1.244	1.262	1.335	1.244	0.031	0.407
	ADFI	0.094	0.104	0.105	0.099	0.003	0.211
	ADG	0.064	0.063	0.067	0.062	0.002	0.357
	FCR	1.462^b^	1.656^a^	1.553^ab^	1.611^a^	0.030	0.016
29–40 d	FBW	2.338	2.313	2.491	2.301	0.049	0.323
	ADFI	0.145	0.148	0.158	0.145	0.005	0.493
	ADG	0.091	0.087	0.096	0.088	0.002	0.289
	FCR	1.591	1.690	1.638	1.643	0.043	0.623
0–40 d	ADFI	0.086	0.092	0.096	0.090	0.002	0.215
	ADG	0.058	0.057	0.061	0.057	0.002	0.428
	FCR	1.499	1.631	1.569	1.588	0.033	0.152
Female							
0–14 d	IBW	0.043	0.044	0.044	0.044	< 0.001	0.754
	FBW	0.340	0.361	0.367	0.346	0.007	0.206
	ADFI	0.029^b^	0.032^a^	0.031^ab^	0.030^ab^	0.001	0.083
	ADG	0.021	0.023	0.023	0.021	0.001	0.114
	FCR	1.362	1.370	1.322	1.396	0.019	0.175
15–28 d	FBW	1.195	1.194	1.236	1.215	0.029	0.796
	ADFI	0.090	0.094	0.096	0.096	0.002	0.116
	ADG	0.061	0.059	0.062	0.062	0.002	0.725
	FCR	1.480	1.588	1.551	1.562	0.043	0.474
29–40 d	FBW	2.235	2.194	2.334	2.280	0.047	0.288
	ADFI	0.137^b^	0.140^b^	0.153^a^	0.147^ab^	0.003	0.049
	ADG	0.087^ab^	0.083^b^	0.092^a^	0.089^ab^	0.002	0.080
	FCR	1.588	1.685	1.666	1.651	0.038	0.536
0–40 d	ADFI	0.083^b^	0.086^ab^	0.090^a^	0.088^a^	0.001	0.036
	ADG	0.055^ab^	0.054^b^	0.057^a^	0.056^ab^	0.001	0.140
	FCR	1.517	1.605	1.583	1.585	0.021	0.114

*Con(+) *The normal-protein control group, *Con(-) *The low-protein control group, *M&C(-) *The methionine and cysteine restriction group, *M&C(+) *The methionine and dysteine supplementation group, *IBW *Initial body weight, *FBW *Final body weight, *ADFI *Average daily feed intake, *ADG *Average daily gain, *FCR *Feed:Gain, *SEM* Standard error of the mean

^a,b^Dissimilar letters represent significant difference among different treatments (*P* < 0.05)

#### BCAA allowance

There were significant differences in BW, ADFI, ADG, and FCR among the four treatments at different phases in male and female broilers. However, no significant differences were observed between the BCAA supplementation and restriction groups at each phase. Broilers fed diets with adjusted BCAA concentrations exhibited poorer performance than those in the control group (Table 6).

**Table 6:** Effects of different BCAA allowance on growth performance of AA broilers

**Item**		**Con(+)**	**Con(-)**	**BCAA(-)**	**BCAA(+)**	**SEM**	***P*-value**
Male							
0–14 d	IBW	0.044	0.043	0.043	0.043	< 0.001	0.361
	FBW	0.347^b^	0.383^a^	0.339^b^	0.352^b^	0.004	< 0.001
	ADFI	0.029^b^	0.034^a^	0.029^b^	0.030^b^	0.001	0.006
	ADG	0.022^b^	0.025^a^	0.021^b^	0.022^b^	0.001	0.018
	FCR	1.304	1.363	1.384	1.350	0.022	0.171
15–28 d	FBW	1.244^a^	1.262^a^	1.128^b^	1.107^b^	0.009	< 0.001
	ADFI	0.094^b^	0.104^a^	0.093^b^	0.096^b^	0.001	< 0.001
	ADG	0.064^a^	0.063^a^	0.056^b^	0.054^b^	0.001	< 0.001
	FCR	1.462^c^	1.656^b^	1.658^b^	1.773^a^	0.027	< 0.001
29–40 d	FBW	2.338^a^	2.313^a^	2.195^b^	2.100^c^	0.019	< 0.001
	ADFI	0.145^a^	0.148^a^	0.143^ab^	0.139^b^	0.002	0.033
	ADG	0.091^a^	0.087^ab^	0.089^ab^	0.083^b^	0.002	0.076
	FCR	1.591	1.690	1.608	1.680	0.034	0.189
0–40 d	ADFI	0.086^b^	0.092^a^	0.085^b^	0.086^b^	< 0.001	< 0.001
	ADG	0.058^a^	0.057^a^	0.054^ab^	0.051^b^	0.001	0.033
	FCR	1.499^b^	1.631^a^	1.585^ab^	1.670^a^	0.032	0.031
Female							
0–14 d	IBW	0.043	0.044	0.044	0.044	< 0.001	0.751
	FBW	0.340^bc^	0.361^a^	0.336^c^	0.358^ab^	0.006	0.040
	ADFI	0.029	0.032	0.028	0.031	0.001	0.130
	ADG	0.021	0.023	0.021	0.023	0.001	0.136
	FCR	1.362	1.370	1.335	1.359	0.034	0.946
15–28 d	FBW	1.195^a^	1.194^a^	1.160^ab^	1.150^b^	0.009	0.046
	ADFI	0.090	0.094	0.094	0.095	0.002	0.298
	ADG	0.061^a^	0.059^ab^	0.059^ab^	0.057^b^	0.001	0.069
	FCR	1.480^b^	1.588^a^	1.606^a^	1.670^a^	0.024	0.005
29–40 d	FBW	2.235^a^	2.194^ab^	2.169^ab^	2.109^b^	0.023	0.053
	ADFI	0.137	0.140	0.140	0.137	0.003	0.794
	ADG	0.087^a^	0.083^ab^	0.084^ab^	0.080^b^	0.001	0.057
	FCR	1.585	1.685	1.668	1.722	0.032	0.143
0–40 d	ADFI	0.083	0.086	0.085	0.085	0.001	0.259
	ADG	0.055^a^	0.054^ab^	0.053^ab^	0.051^b^	0.001	0.158
	FCR	1.517^b^	1.605^ab^	1.592^ab^	1.657^a^	0.029	0.079

*Con(+) *The normal-protein control group, *Con(-) *The low-protein control group, *BCAA(-) *The BCAA restriction group, *BCAA(+) *The BCAA supplementation group, *IBW *Initial body weight, *FBW *Final body weight, *ADFI *Average daily feed intake, *ADG *Average daily gain, *FCR *Feed:Gain, *SEM* Standard error of the mean

^a−c^Dissimilar letters represent significant difference among different treatments (*P* < 0.05)

#### Threonine allowance

There were significant differences in BW, ADFI, ADG, and FCR among the four treatments at different phases in male and female broilers, with the threonine supplementation group showing the worst performance. In addition, broilers that were fed diets with adjusted threonine concentrations exhibited poorer performance than those in the control groups but the threonine restriction group performed slightly better than the threonine supplementation group (Table 7).

**Table 7:** Effects of different threonine allowance on growth performance of AA broilers

**Item**		**Con(+)**	**Con(-)**	**Thr(-)**	**Thr(+)**	**SEM**	***P*-value**
Male							
0–14 d	IBW	0.044	0.043	0.044	0.044	< 0.001	0.341
	FBW	0.347^b^	0.383^a^	0.378^a^	0.345^b^	0.005	0.001
	ADFI	0.029^b^	0.034^a^	0.033^a^	0.029^b^	0.001	0.001
	ADG	0.022^b^	0.025^a^	0.024^a^	0.021^b^	< 0.001	0.006
	FCR	1.304	1.363	1.377	1.363	0.028	0.395
15–28 d	FBW	1.244^a^	1.262^a^	1.206^a^	1.073^b^	0.020	0.002
	ADFI	0.094^b^	0.104^a^	0.099^a^	0.088^c^	0.001	0.001
	ADG	0.064^a^	0.063^ab^	0.059^b^	0.052^c^	0.001	0.001
	FCR	1.462^b^	1.656^a^	1.679^a^	1.686^a^	0.022	0.001
29–40 d	FBW	2.338^a^	2.313^a^	2.280^a^	2.005^b^	0.027	< 0.001
	ADFI	0.145^a^	0.148^a^	0.151^a^	0.134^b^	0.003	0.008
	ADG	0.091^a^	0.087^a^	0.090^a^	0.078^b^	0.002	0.006
	FCR	1.591	1.690	1.685	1.727	0.036	0.179
0–40 d	ADFI	0.086^b^	0.092^a^	0.091^a^	0.081^c^	0.001	< 0.001
	ADG	0.058^a^	0.057^a^	0.056^a^	0.049^b^	0.001	0.007
	FCR	1.499^b^	1.631^a^	1.632^a^	1.654^a^	0.029	0.029
Female							
0–14 d	IBW	0.043	0.044	0.043	0.043	< 0.001	0.559
	FBW	0.340^b^	0.361^a^	0.336^b^	0.325^b^	0.006	0.018
	ADFI	0.029^b^	0.032^a^	0.029^ab^	0.028^b^	0.001	0.030
	ADG	0.021^ab^	0.023^a^	0.021^b^	0.020^b^	0.001	0.046
	FCR	1.362	1.370	1.425	1.377	0.023	0.358
15–28 d	FBW	1.195^a^	1.194^a^	1.115^b^	1.084^b^	0.009	< 0.001
	ADFI	0.090	0.094	0.090	0.087	0.002	0.177
	ADG	0.061^a^	0.059^a^	0.056^b^	0.054^b^	0.001	0.004
	FCR	1.480^b^	1.588^a^	1.613^a^	1.606^a^	0.023	0.019
29–40 d	FBW	2.235^a^	2.194^a^	2.077^b^	1.986^b^	0.026	0.001
	ADFI	0.137^a^	0.140^a^	0.137^a^	0.128^b^	0.002	0.038
	ADG	0.087^a^	0.083^ab^	0.080^b^	0.075^c^	0.001	0.001
	FCR	1.585^b^	1.685^a^	1.708^a^	1.707^a^	0.021	0.061
0–40 d	ADFI	0.083^a^	0.086^a^	0.083^a^	0.079^b^	0.001	0.019
	ADG	0.055^a^	0.054^a^	0.051^b^	0.049^c^	0.001	0.001
	FCR	1.517^b^	1.605^a^	1.625^a^	1.618^a^	0.019	0.026

*Con(+)* The normal-protein control group, *Con(-) *The low-protein control group, *Thr(-) *The threonine restriction group, *Thr(+) *The threonine supplementation group, *IBW *Initial body weight, *FBW *Final body weight, *ADFI *Average daily feed intake, *ADG *Average daily gain, *FCR *Feed:Gain, *SEM* Standard error of the mean

^a−c^Dissimilar letters represent significant difference among different treatments (*P* < 0.05)

#### Tryptophan allowance

There were significant differences among the four treatments in all indices at the starter and grower phases and in BW at the finisher phase in male broilers. The tryptophan-restricted group showed the worst performance during the entire lifespan. There was no significant difference between the tryptophan-supplemented and low-protein control groups; however, the tryptophan-supplemented group showed an upward trend during the finisher phase. There were differences among the four treatments in FCR at different phases, and in BW and ADFI at the starter phase in female broilers. Except for the normal protein control group, the tryptophan-supplemented group had the lowest FCR in all phases (Table 8).

**Table 8:** Effects of different tryptophan allowance on growth performance of AA broilers

**Item**		**Con(+)**	**Con(-)**	**Trp(-)**	**Trp(+)**	**SEM**	***P*-value**
Male							
0–14 d	IBW	0.044	0.043	0.043	0.043	< 0.001	0.217
	FBW	0.347^b^	0.383^a^	0.344^b^	0.368^a^	0.005	0.004
	ADFI	0.029^b^	0.034^a^	0.029^b^	0.032^a^	0.001	0.002
	ADG	0.022^bc^	0.025^a^	0.021^c^	0.023^ab^	0.001	0.015
	FCR	1.304	1.363	1.364	1.351	0.022	0.301
15–28 d	FBW	1.244^a^	1.262^a^	1.112^b^	1.255^a^	0.013	< 0.001
	ADFI	0.094^b^	0.104^a^	0.092^b^	0.100^a^	0.002	0.012
	ADG	0.064^a^	0.063^a^	0.055^b^	0.063^a^	0.001	< 0.001
	FCR	1.462^b^	1.656^a^	1.675^a^	1.586^ab^	0.040	0.026
29–40 d	FBW	2.338^a^	2.313^a^	2.122^b^	2.349^a^	0.015	< 0.001
	ADFI	0.145	0.148	0.140	0.150	0.003	0.343
	ADG	0.091^a^	0.087^ab^	0.084^b^	0.091^ab^	0.002	0.130
	FCR	1.591	1.690	1.660	1.649	0.034	0.285
0–40 d	ADFI	0.086^b^	0.092^a^	0.084^b^	0.091^a^	0.001	< 0.001
	ADG	0.058^a^	0.057^a^	0.052^b^	0.057^a^	0.001	0.019
	FCR	1.499^b^	1.631^a^	1.623^a^	1.591^ab^	0.029	0.048
Female							
0–14 d	IBW	0.043	0.044	0.044	0.044	< 0.001	0.503
	FBW	0.340^b^	0.361^a^	0.355^ab^	0.346^ab^	0.005	0.078
	ADFI	0.029	0.032	0.031	0.029	0.001	0.053
	ADG	0.021	0.023	0.022	0.021	0.001	0.441
	FCR	1.362	1.370	1.383	1.348	0.027	0.867
15–28 d	FBW	1.195	1.194	1.192	1.177	0.012	0.773
	ADFI	0.090^b^	0.094^ab^	0.098^a^	0.094^ab^	0.001	0.048
	ADG	0.061	0.059	0.060	0.059	0.001	0.832
	FCR	1.480^b^	1.588^ab^	1.640^a^	1.590^ab^	0.033	0.054
29–40 d	FBW	2.235	2.194	2.190	2.202	0.026	0.677
	ADFI	0.137	0.140	0.141	0.144	0.002	0.412
	ADG	0.087	0.083	0.083	0.085	0.001	0.317
	FCR	1.585	1.685	1.702	1.683	0.026	0.121
0–40 d	ADFI	0.083	0.086	0.088	0.086	0.001	0.112
	ADG	0.055	0.054	0.054	0.054	0.001	0.831
	FCR	1.517^b^	1.605^a^	1.633^a^	1.585^ab^	0.022	0.066

*Con(+) *The normal-protein control group, *Con(-) *The low-protein control group, *Trp(-) *The tryptophan restriction group, *Trp(+) *The tryptophan supplementation group, *IBW *Initial body weight, *FBW *Final body weight, *ADFI *Average daily feed intake, *ADG *Average daily gain, *FCR *Feed:Gain, *SEM* Standard error of the mean

^a−c^Dissimilar letters represent significant difference among different treatments (*P* < 0.05)

In general, the growth performance of male and female broilers was similar; however, the differences among females were less significant.

### Carcass characteristic

Male and female broilers had the same carcass characteristics and are therefore described uniformly in the following paragraphs.

#### Lysine and arginine allowance

There were significant differences in all indices except the thigh rate (TR) among the four treatments. The lysine- and arginine-restricted groups had the lowest dressing rate (DR), half-evisceration rate (HR), evisceration rate (ER), and highest fat pad rate (FR). However, the lysine and arginine supplementation group was significantly higher than the low-protein control group in breast rate (BR) (Tables 9 and 10).

**Table 9:** Effects of amino acid combinations with different patterns on carcass characteristic of AA broilers (male)^1^

Item	DR, %	HR, %	ER, %	FR, %	BR, %	TR, %
Lys&Arg						
Con(+)	94.181^ab^	88.647^a^	76.291^a^	2.275^b^	28.977^a^	19.962
Con(-)	94.249^a^	88.740^a^	76.220^a^	2.294^b^	25.423^b^	21.103
L&A(-)	92.680^b^	87.013^b^	74.116^b^	2.579^a^	27.631^ab^	20.825
L&A(+)	93.314^ab^	88.111^ab^	75.629^ab^	2.455^ab^	29.306^a^	20.057
SEM	0.337	0.393	0.513	0.071	0.851	0.551
* P*-value	0.018	0.046	0.030	0.020	0.019	0.433
Met&Cys						
Con(+)	94.181	88.647	76.291	2.275^b^	28.977^a^	19.962
Con(-)	94.249	88.740	76.220	2.294^b^	25.423^b^	21.103
M&C(-)	94.192	88.614	75.228	2.859^a^	27.880^ab^	20.696
M&C(+)	93.857	87.935	74.888	2.985^a^	24.777^b^	20.510
SEM	0.339	0.432	0.535	0.119	1.037	0.602
* P*-value	0.873	0.618	0.213	0.001	0.034	0.662
BCAA						
Con(+)	94.181	88.647	76.291^a^	2.275^b^	28.977^a^	19.962
Con(-)	94.249	88.740	76.220^a^	2.294^b^	25.423^b^	21.103
BCAA(-)	93.616	87.677	74.711^b^	3.034^a^	24.660^b^	21.375
BCAA(+)	93.694	87.862	74.086^b^	3.080^a^	23.649^b^	20.827
SEM	0.294	0.355	0.415	0.084	1.009	0.514
* P*-value	0.428	0.202	0.004	< 0.001	0.009	0.300
Thr						
Con(+)	94.181	88.647	76.291	2.275^b^	28.977^a^	19.962
Con(-)	94.249	88.740	76.220	2.294^b^	25.423^b^	21.103
Thr(-)	92.946	87.509	74.947	2.452^a^	24.537^b^	19.897
Thr(+)	93.700	87.868	74.724	2.574^a^	24.391^b^	20.245
SEM	0.548	0.455	0.655	0.06	0.871	0.708
* P*-value	0.518	0.257	0.256	0.008	0.003	0.709
Trp						
Con(+)	94.181^a^	88.647	76.291^a^	2.275^b^	28.977^a^	19.962^ab^
Con(-)	94.249^a^	88.740	76.220^a^	2.294^b^	25.423^b^	21.103^ab^
Trp(-)	94.063^a^	88.137	74.819^b^	3.044^a^	25.375^b^	19.263^b^
Trp(+)	92.561^b^	87.520	74.339^b^	2.458^b^	24.381^b^	21.806^a^
SEM	0.326	0.421	0.386	0.109	0.980	0.624
* P*-value	0.008	0.258	0.009	< 0.001	0.020	0.079

*Con(+) *The normal-protein control group, *Con(-) *The low-protein control group, *L&A(-) *The lysine and arginine restriction group, *L&A(+) *The lysine and arginine supplementation group, *M&C(-) *The methionine and cysteine restriction group, *M&C(+) *The methionine and cysteine supplementation group, *BCAA(-) *The BCAA restriction group, *BCAA(+) *The BCAA supplementation group, *Thr(-) *The threonine restriction group, *Thr(+) *The threonine supplementation group, *Trp(-) *The tryptophan restriction group, *Trp(+) *The tryptophan supplementation group, *DR *Dressing rate, *HR *Half-eviscerated rate, *ER *Eviscerated rate, *FR *Fat pad rate, *BR *Breast rate, *TR *Thigh rate, *SEM* Standard error of the mean

^1^DR = Dressed weight/live body weight, HR = Half-eviscerated weight/live body weight, ER = Eviscerated weight/live body weight, FR = Fat pad weight/dressed weight, BR = Breast weight/eviscerated weight, TR = Thigh weight/eviscerated weight

^a,b^Dissimilar letters represent significant difference among different treatments (*P* < 0.05)

**Table 10:** Effects of amino acid combinations with different patterns on carcass characteristic of AA broilers (female)^1^

Item	DR, %	HR, %	ER, %	FR, %	BR, %	TR, %
Lys&Arg						
Con(+)	93.478	87.901	75.945	2.275^b^	27.737^a^	19.796^a^
Con(-)	93.712	88.142	75.837	2.558^ab^	25.809^b^	19.668^ab^
L&A(-)	93.031	87.581	75.164	2.918^a^	26.891^ab^	18.733^b^
L&A(+)	93.652	88.411	76.155	2.942^a^	26.705^ab^	19.658^ab^
SEM	0.313	0.389	0.514	0.116	0.413	0.318
* P*-value	0.453	0.538	0.594	0.007	0.037	0.102
Met&Cys						
Con(+)	93.478	87.901	75.945	2.275^b^	27.737^a^	19.796
Con(-)	93.712	88.142	75.837	2.558^b^	25.809^b^	19.668
M&C(-)	93.302	87.691	75.534	3.090^a^	25.851^ab^	20.079
M&C(+)	92.246	87.354	74.784	3.176^a^	24.121^b^	20.556
SEM	0.593	0.427	0.589	0.115	0.608	0.378
* P*-value	0.598	0.639	0.518	< 0.001	0.008	0.417
BCAA						
Con(+)	93.478	87.901	75.945	2.275^b^	27.737^a^	19.796
Con(-)	93.712	88.142	75.837	2.558^b^	25.809^b^	19.668
BCAA(-)	93.315	87.264	74.985	3.096^a^	24.629^bc^	20.945
BCAA(+)	93.751	88.22	75.267	3.239^a^	23.713^c^	20.819
SEM	0.307	0.431	0.549	0.112	0.487	0.502
* P*-value	0.725	0.420	0.584	< 0.001	< 0.001	0.315
Thr						
Con(+)	93.478	87.901^ab^	75.945^a^	2.275^b^	27.737^a^	19.796
Con(-)	93.712	88.142^ab^	75.837^a^	2.558^b^	25.809^ab^	19.668
Thr(-)	93.614	88.335^a^	75.387^ab^	3.199^a^	23.690^bc^	19.671
Thr(+)	92.996	86.958^b^	74.095^b^	3.041^a^	22.847^c^	20.102
SEM	0.297	0.402	0.489	0.112	0.658	0.539
* P*-value	0.369	0.127	0.077	< 0.001	0.001	0.946
Trp						
Con(+)	93.478^ab^	87.901^ab^	75.945^ab^	2.275^c^	27.737^a^	19.796
Con(-)	93.712^ab^	88.142^ab^	75.837^ab^	2.558^bc^	25.809^b^	19.668
Trp(-)	94.016^a^	89.150^a^	76.084^a^	3.194^a^	23.823^c^	19.568
Trp(+)	92.772^b^	86.802^b^	74.376^b^	2.89^ab^	25.077^b^	18.841
SEM	0.345	0.568	0.531	0.123	0.402	0.414
* P*-value	0.118	0.074	0.151	0.003	< 0.001	0.429

*Con(+) *The normal-protein control group, *Con(-) *The low-protein control group, *L&A(-) *The lysine and arginine restriction group, *L&A(+) *The lysine and arginine supplementation group, *M&C(-) *The methionine and cysteine restriction group, *M&C(+) *The methionine and cysteine supplementation group, *BCAA(-) *The BCAA restriction group, *BCAA(+) *The BCAA supplementation group, *Thr(-)* The threonine restriction group, *Thr(+) *The threonine supplementation group, *Trp(-) *The tryptophan restriction group, *Trp(+) *The tryptophan supplementation group, *DR *Dressing rate, *HR *Half-eviscerated rate, *ER *Eviscerated rate, *FR *Fat pad rate, *BR *Breast rate, *TR *Thigh rate, *SEM* Standard error of the mean

^1^DR = Dressed weight/live body weight, HR = Half-eviscerated weight/live body weight, ER = Eviscerated weight/live body weight, FR = Fat pad weight/dressed weight, BR = Breast weight/eviscerated weight, TR = Thigh weight/eviscerated weight

^a−c^Dissimilar letters represent significant difference among different treatments (*P* < 0.05)

#### Methionine and cysteine allowance

There were significant differences in the FR and BR among the four treatments. FR was higher in the methionine and cysteine restriction groups and the methionine and cysteine supplementation groups than in the normal-protein control and low-protein control groups, whereas BR was not different from the low-protein control group (Tables 9 and 10).

#### BCAA allowance

There were significant differences in ER, FR, and BR among the four treatments. Both the BCAA restriction group and the BCAA supplementation group were lower than the low-protein control and the normal-protein control groups in ER and higher in FR, but there was no difference in BR compared to the low-protein control group (Tables 9 and 10).

#### Threonine allowance

There were significant differences in FR and BR among the four treatments, with both the threonine-restriction and threonine-supplementation groups having significantly higher FR and lower BR values than the other 2 groups (Tables 9 and 10).

#### Tryptophan allowance

There were significant differences among the four treatments in ER, FR, and BR, and a trend in TR. Both the tryptophan restriction group and the tryptophan supplementation group had a lower ER than the normal-protein control group and the low-protein control group, and there was a significant difference in the TR between them. In addition, the tryptophan restriction group had the highest FR (Tables 9 and 10).

### Hepatic enzyme activities and serum biochemical parameters

**Fig. 1:**
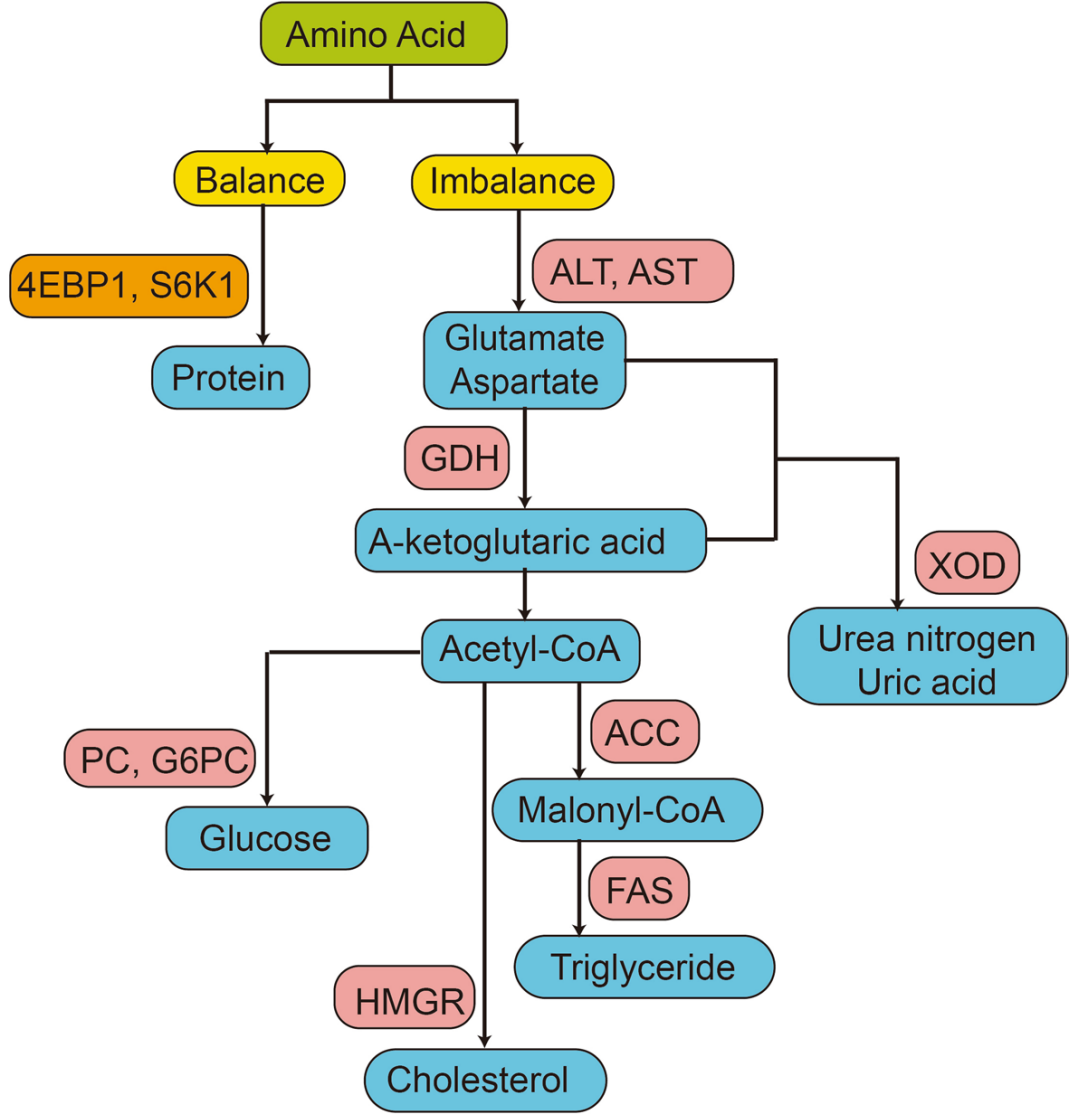
Amino acid metabolism pathways. AST: aspartate aminotransferase; ALT: alanine aminotransferase; TG: Triglyceride; ACC: Acetyl CoA carboxylase; FAS: Fatty acid synthase; GLU: Blood glucose; G6PC: Glucose-6-phosphatase; PC: Pyruvate carboxylase; TC: Cholesterol; HMGR: HMG CoA reductase; XOD: Xanthine oxidase; BUN: Urea nitrogen; UA: Uric acid; S6K1: The 70-kDa ribosomal protein S6 kinase; 4EBP1: Eukaryotic translation initiation factor 4E-binding protein-1

Hepatic enzyme activities and serum biochemical parameters of broilers were determined according to amino acid metabolic pathways (Fig. 1).

**Fig. 2:**
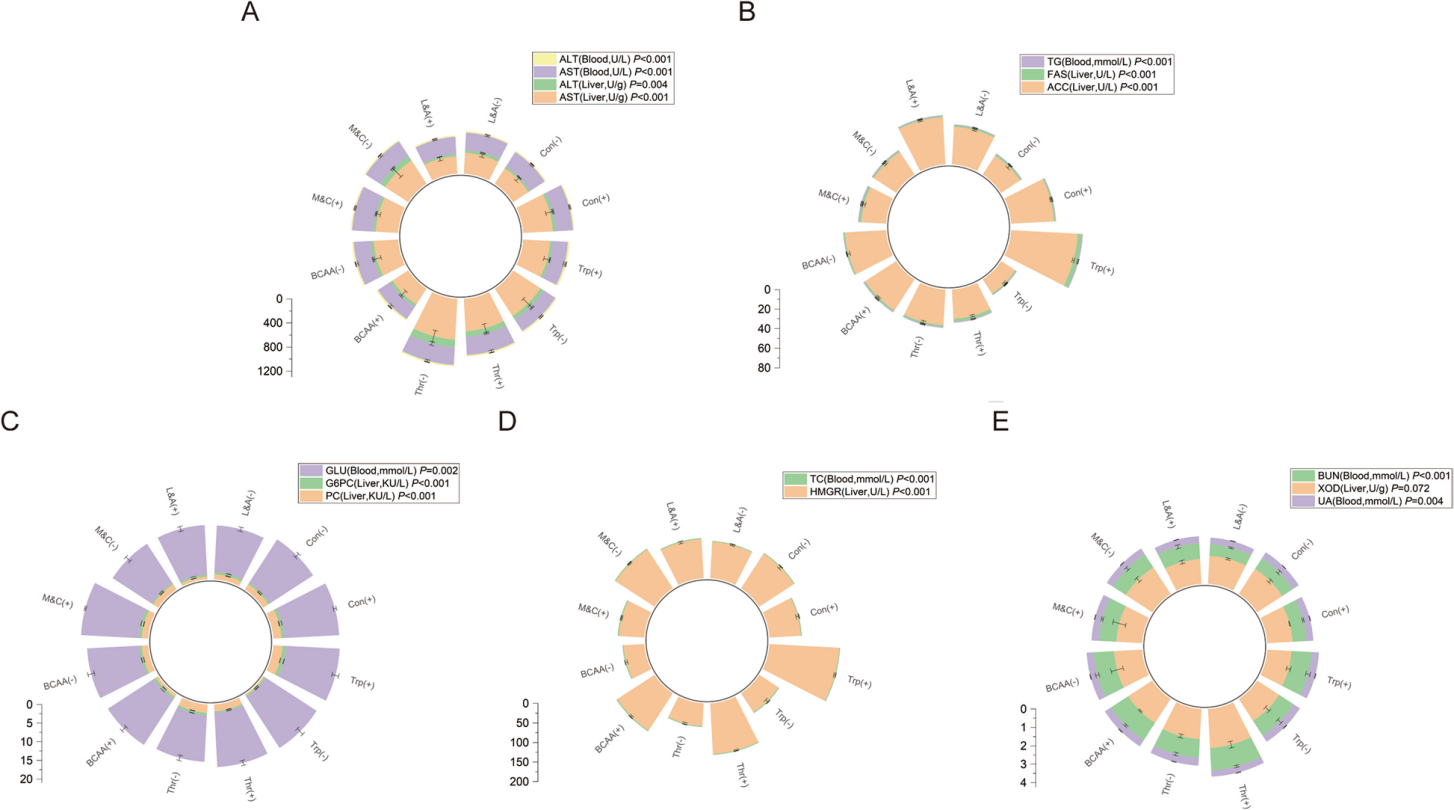
Effects of amino acid combinations with different patterns on hepatic enzyme activities and serum biochemical parameters of AA broilers (male). **A** Transamination pathway; **B** Fat synthesis pathway; **C** Gluconeogenic pathway; **D** Cholesterol synthesis pathway; **E** Uric acid excretion pathway. Con(+): The normal-protein control group; Con(-): The low-protein control group; L&A(-): The lysine and arginine restriction group; L&A(+): The lysine and arginine supplementation group; M&C(-): The methionine and cysteine restriction group; M&C(+): The methionine and cysteine supplementation group; BCAA(-): The BCAA restriction group; BCAA(+): The BCAA supplementation group; Thr(-): The threonine restriction group; Thr(+): The threonine supplementation group; Trp(-): The tryptophan restriction group; Trp(+): The tryptophan supplementation group; AST: Aspartate aminotransferase; ALT: Alanine aminotransferase; TG: Triglyceride; ACC: Acetyl CoA carboxylase; FAS: Fatty acid synthase; GLU: Blood glucose; G6PC: Glucose-6-phosphatase; PC: Pyruvate carboxylase; TC: Cholesterol; HMGR: HMG CoA reductase; XOD: Xanthine oxidase; BUN: Urea nitrogen; UA: Uric acid

There were significant differences in hepatic enzyme activity and serum biochemical parameters among all treatments in male broilers. In the transamination pathway, the threonine restriction group had the highest, whereas the lysine and arginine supplementation groups had the lowest hepatic enzyme activities and serum metabolites (Fig. 2A). The tryptophan supplementation group had the highest, whereas the tryptophan restriction group had the lowest fat synthesis pathway (Fig. 2B). In the gluconeogenesis pathway, the methionine- and cysteine-supplemented groups had the highest levels, and the methionine- and cysteine-restricted groups had the lowest levels (Fig. 2C). Regarding the cholesterol synthesis pathway, the tryptophan supplementation group showed the highest expression, and the BCAA restriction group showed the lowest expression (Fig. 2D). In the Uric acid excretion pathway, the threonine supplementation group had the highest expression, and the normal protein control group had the lowest expression (Fig. 2E).

**Fig. 3:**
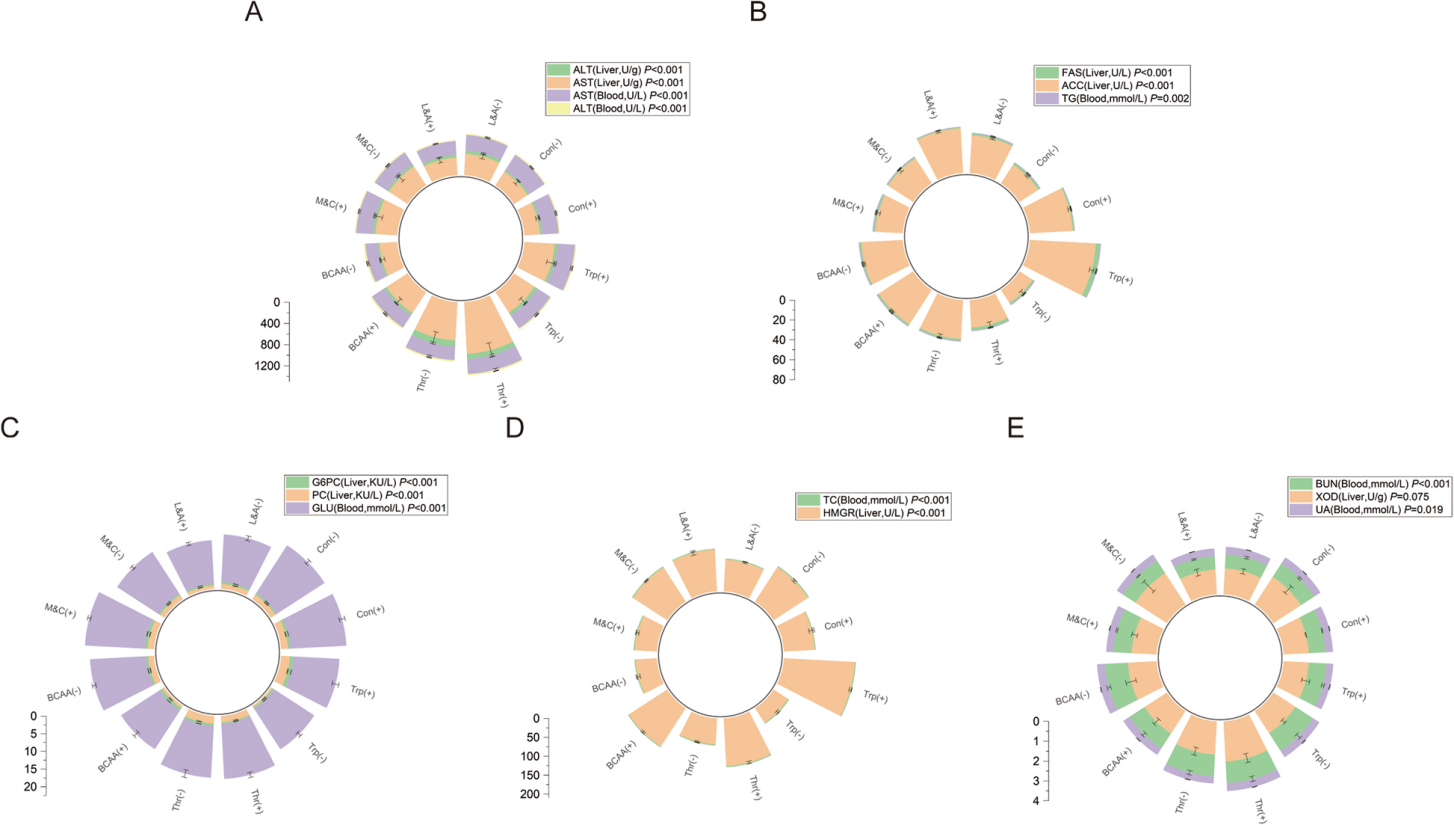
Effects of amino acid combinations with different patterns on hepatic enzyme activities and serum biochemical parameters of AA broilers (female). **A** Transamination pathway; **B** Fat synthesis pathway; **C** Gluconeogenic pathway; **D** Cholesterol synthesis pathway; **E** Uric acid excretion pathway. Con(+): The normal-protein control group; Con(-): The low-protein control group; L&A(-): The lysine and arginine restriction group; L&A(+): The lysine and arginine supplementation group; M&C(-): The methionine and cysteine restriction group; M&C(+): The methionine and cysteine supplementation group; BCAA(-): The BCAA restriction group; BCAA(+): The BCAA supplementation group; Thr(-): The threonine restriction group; Thr(+): The threonine supplementation group; Trp(-): The tryptophan restriction group; Trp(+): The tryptophan supplementation group; AST: Aspartate aminotransferase; ALT: Alanine aminotransferase; TG: Triglyceride; ACC: Acetyl CoA carboxylase; FAS: Fatty acid synthase; GLU: Blood glucose; G6PC: Glucose-6-phosphatase; PC: Pyruvate carboxylase; TC: Cholesterol; HMGR: HMG CoA reductase; XOD: Xanthine oxidase; BUN: Urea nitrogen; UA: Uric acid

In female broilers, variations in hepatic enzyme activity among the different treatment groups were fundamentally similar to those observed in males. In the transamination pathway, the threonine supplementation group had the highest, whereas the BCAA restriction group had the lowest hepatic enzyme activities and serum metabolites (Fig. 3A). The tryptophan supplementation group had the highest and the tryptophan restriction group had the lowest fat synthesis pathway (Fig. 3B). In the gluconeogenesis pathway, the methionine- and cysteine-supplemented groups had the highest values, while the lysine-and arginine-supplemented groups had the lowest values (Fig. 3C). In the Cholesterol synthesis pathway, the tryptophan supplementation group showed the highest expression, and the tryptophan restriction group showed the lowest expression (Fig. 3D). In the Uric acid excretion pathway, the threonine supplementation group had the highest expression and the lysine and arginine supplementation groups had the lowest expression (Fig. 3E).

### Expressions of mRNA in breast muscle

**Fig. 4:**
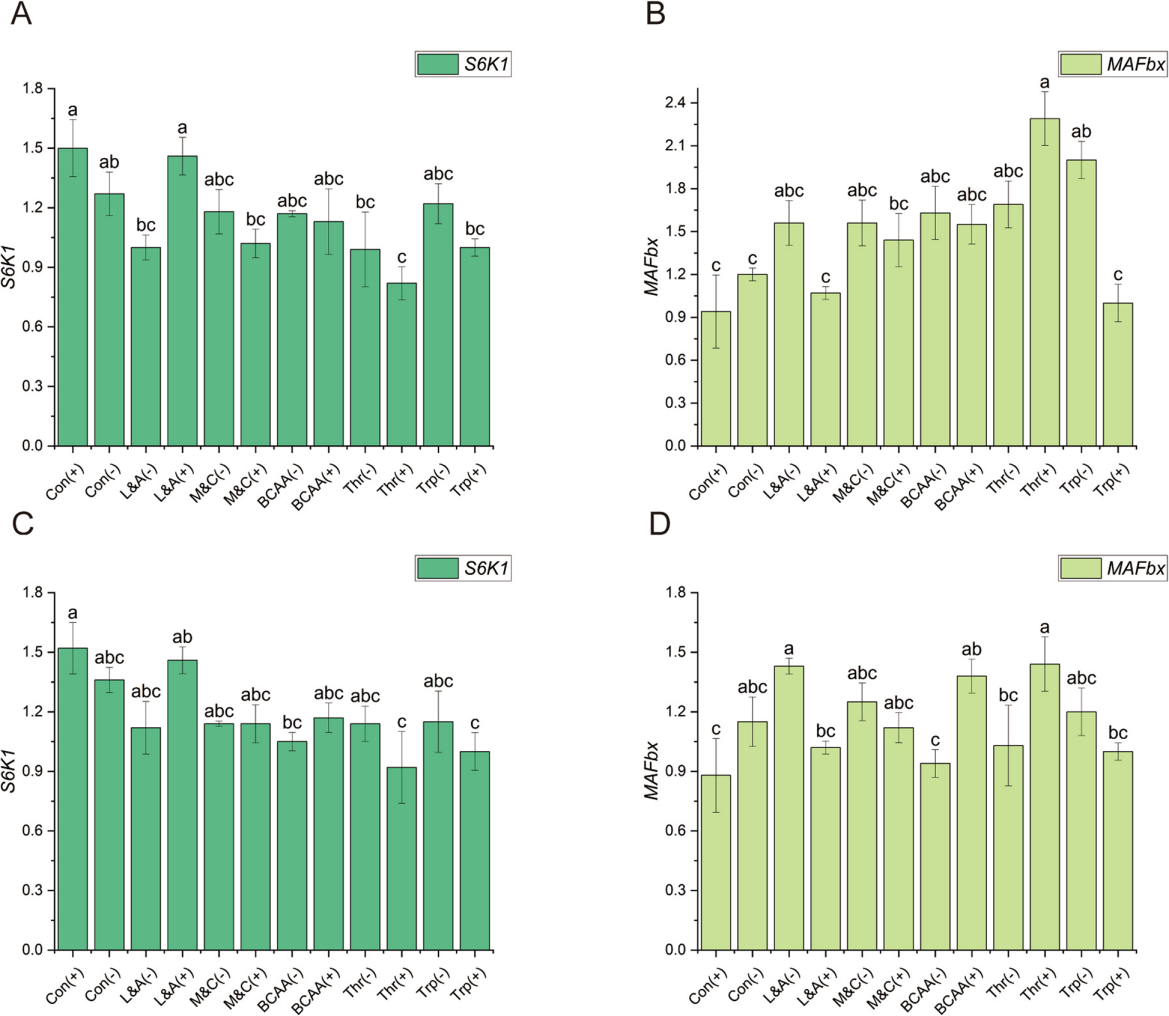
Effects of amino acid combinations with different patterns on alterations of mRNA expressions in the breast muscle of AA broilers. **A** Male-*S6K1*; **B** Male-*MAFbx*; **C** Femal-*S6K1*; **D** Female-*MAFbx*. Con(+): The normal-protein control group; Con(-): The low-protein control group; L&A(-): The lysine and arginine restriction group; L&A(+): The lysine and arginine supplementation group; M&C(-): The methionine and cysteine restriction group; M&C(+): The methionine and cysteine supplementation group; BCAA(-): The BCAA restriction group; BCAA(+): The BCAA supplementation group; Thr(-): The threonine restriction group; Thr(+): The threonine supplementation group; Trp(-): The tryptophan restriction group; Trp(+): The tryptophan supplementation group. *S6K1*: The 70-kDa ribosomal protein S6 kinase; *MAFbx*: Muscle atrophy f-box. ^a–c^Dissimilar letters represent significant difference among different treatments (*P* < 0.05)

There were significant differences in *S6K1* and *MAFbx* expression but no significant differences in the other indices between male (Fig. 4) and female broilers (Fig. 4 and S1). The threonine supplementation group had significantly lower levels than the other treatments, and the normalized protein control group and the lysine and arginine supplementation groups had the highest levels of *S6K1*. The threonine supplementation group had significantly higher *MAFbx* than the other treatments, and the lysine and arginine supplementation groups had the lowest *MAFbx*.

### Hepatic untargeted metabolomics analysis

Based on the screening of the data in Figs. 2, 3 and 4, as well as the sex-specific sensitivity of animals to amino acid balance. Male broiler chickens were used as models. The normal protein control group was fed a normal protein diet (NP), the low-protein control group was fed a low-protein diet (LP), the lysine and arginine supplementation group was fed a low-protein amino acid-balanced diet (LPAB), and the threonine supplementation group was fed a low-protein amino acid-imbalanced diet (LPAI). These diets were subjected to untargeted metabolomic analysis to identify amino acid balance biomarkers.

#### Principal component analysis

**Fig. 5:**
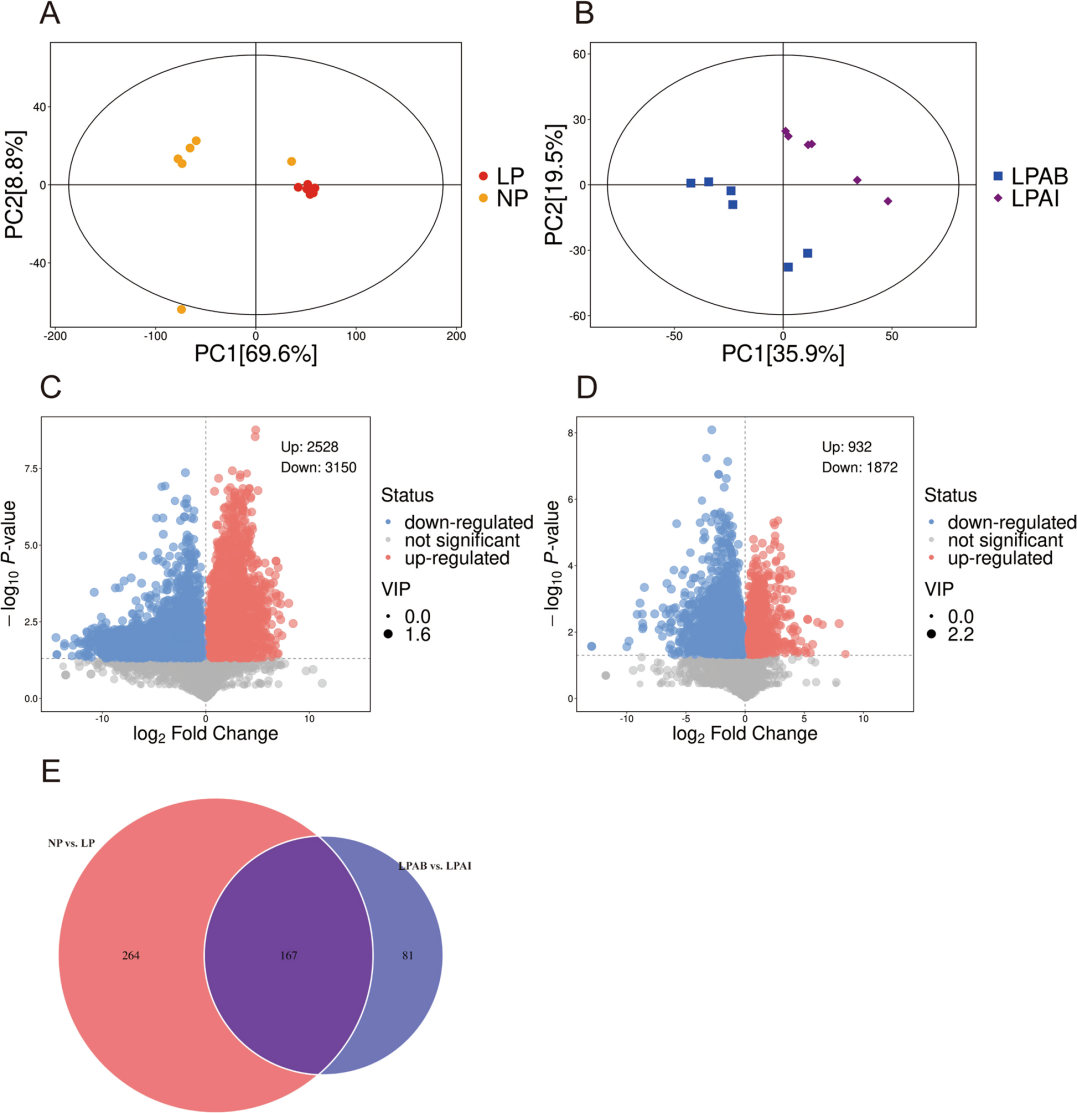
Principal component (PC) analysis of NP vs. LP liver metabolomics data (**A**) and LPAB vs. LPAI liver metabolomics data (**B**). Volcano of NP vs. LP liver differential metabolites (**C**) and LPAB vs. LPAI liver differential metabolites (**D**). Venn plot of NP vs. LP and LPAB vs. LPAI shared liver differential metabolites (**E**). NP: Normal CP diet; LP: Low-CP diet; LPAB: Low-CP amino acid balance diet; LPAI: Low-CP amino acid imbalance diet

The PCA score plots revealed a significant separation between the NP and LP, with PC1 and PC2 accounting for 69.6% and 8.8% of the sample variation, respectively (Fig. 5A). Similarly, there was a notable distinction between LPAB and LPAI, as shown in the PCA score plots, where PC1 and PC2 explained 35.9% and 19.5% of sample variation, respectively (Fig. 5B).

#### Orthogonal partial least-squares discriminant analysis

The OPLS-DA score plot demonstrated a significant separation between the NP and LP, with the abscissa and ordinate explaining intragroup differences, accounting for 40.4% and 17.2% of the sample variation, respectively (Fig. S1A). A permutation test (*n* = 100) was performed to validate the OPLS-DA model. The blue bands depict the original samples, where a more pronounced distribution toward the right indicates more substantial data separation between the groups. The R^2^Y(cum) and Q^2^(cum) values were 0.988 and 0.823, respectively (Fig. S1C), confirming the reliability of these results.

Similarly, the OPLS-DA score plot exhibited a significant separation between LPAB and LPAI, with inter-group differences in the abscissa and ordinate accounting for 21% and 21.6% of the sample variation, respectively (Fig. S1B). The R^2^Y(cum) value was calculated to be 0.994, whereas the Q^2^(cum) value reached 0.857 (Fig. S1D), indicating the high reliability of these results.

#### Identification of significant differential metabolites

Volcano plots revealed 2,528 upregulated metabolites and 3,150 downregulated metabolites between the NP and LP groups, resulting in the identification of 431 differential metabolites (Fig. 5C). Additionally, 932 upregulated and 1,872 downregulated metabolites were detected between LPAB and LPAI based on volcano plot analysis, leading to the final identification of 248 differential metabolites (Fig. 5D). Overall, 167 differential metabolites were identified in the four treatments (Fig. 5E).

#### Acquisition of specific potential biomarkers

To identify the relevant differential metabolites among more than 100 metabolites, the following criteria were used: *P* < 0.05, VIP > 1, and fold-change > 2 or < 0.5. A total of 43 metabolites met the inclusion criteria, in addition, uric acid and urea nitrogen were counted as common amino acid metabolites (Table 11). In broiler production, certain technical procedures such as handling, and blood sampling can induce stress in animals and potentially interfere with in vivo biochemical indices. Therefore, based on the identified differential metabolites, we selected those that exhibited consistent changes in direction and quantitative difference values (QD-value) > 1. The 3 metabolites that met these criteria were Oxypurinol, Pantothenic acid, and D-octopine (Table 12).

**Table 11:** Evaluation score of important shared differential metabolites in four treatments

Item	NP vs. LP			LPAB vs. LPAI		
	VIP	*P*-value^1^	FC^2^	VIP	*P*-value^1^	FC^2^
Oxypurinol^3^	1.522	0.001	0.241	1.987	< 0.001	0.420
Pantothenic acid	1.513	0.002	20.226	2.055	0.001	10.359
D-Octopine	1.529	< 0.001	0.170	1.923	0.003	0.328
Xanthine	1.519	0.003	0.107	1.937	< 0.001	0.348
6-Tuliposide B	1.522	< 0.001	0.188	1.835	< 0.001	0.455
Uridine 5′-monophosphate (UMP)	1.454	< 0.001	8.165	1.800	0.002	2.098
Thiamine	1.352	0.001	0.298	2.028	< 0.001	0.242
N-(4-Hydroxyphenyl)glycine	1.406	0.003	0.202	2.017	< 0.001	0.212
Pyridoxal (Vitamin B_6_)	1.406	0.003	0.202	2.017	< 0.001	0.212
5′-Phosphoribosylglycinamide	1.446	0.022	0.154	2.000	0.013	0.083
Gly-His	1.479	0.004	0.042	2.111	0.004	0.029
1-Palmitoyl-2-arachidonoyl-sn-glycero-3-phosphoserine	1.463	< 0.001	19.560	1.751	0.002	3.283
Chenodeoxycholic acid	1.451	0.007	0.019	1.820	0.032	0.214
Deoxycholic acid	1.451	0.007	0.019	1.820	0.032	0.214
Hyodeoxycholic acid	1.451	0.007	0.019	1.820	0.032	0.214
Isodeoxycholic acid	1.451	0.007	0.019	1.820	0.032	0.214
Ursodeoxycholic acid	1.451	0.007	0.019	1.820	0.032	0.214
Arachidonic acid (AA)	1.356	0.020	0.112	1.827	0.014	0.288
4-Amino-4-deoxychorismate	1.486	< 0.001	0.145	1.922	0.005	0.296
2-Piperidone	1.217	0.030	0.296	1.769	0.015	0.418
D-galacto-Hexodialdose	1.491	0.001	0.085	1.933	0.004	0.096
N-Oleoyl glycine	1.502	0.018	0.009	1.674	0.002	0.065
Linoleoylglycine	1.459	0.009	0.006	1.716	0.006	0.040
1-Stearoyl-2-arachidonoyl-sn-glycero-3-phosphoserine	1.448	< 0.001	11.546	1.767	< 0.001	2.929
5-Tetradecynoic acid	1.487	0.003	0.105	2.010	0.014	0.143
Hygric acid	1.265	0.016	0.186	2.026	0.022	0.091
Pipecolic acid	1.265	0.016	0.186	2.026	0.022	0.091
2-Oxo-4-phosphonobutanoate	1.499	0.004	0.133	1.935	0.008	0.280
M345T195	1.532	< 0.001	0.017	1.850	0.042	0.021
Dephospho-CoA	1.405	0.001	29.684	1.859	< 0.001	7.405
M368T281	1.510	0.003	0.053	2.050	0.012	0.097
Pantetheine 4′-phosphate	1.309	0.002	21.895	1.985	0.008	14.229
Eglumegad	1.485	0.003	0.277	1.878	0.004	0.438
N-Arachidonylglycine	1.422	0.012	0.009	1.795	0.008	0.035
N-Acetylmannosamine	1.502	0.001	0.219	1.897	0.005	0.353
Pyridoxamine	1.484	0.001	0.205	1.834	0.001	0.267
Goralatide (acetate)	1.491	0.004	0.056	2.103	0.004	0.090
N-Acetylglucosaminylasparagine	1.524	< 0.001	6.893	1.900	< 0.001	3.925
3-beta-D-Galactosyl-sn-glycerol	1.406	0.007	0.007	1.985	0.015	0.026
M730T215	1.413	0.001	17.590	1.730	0.023	15.166
Acetyl-CoA	1.365	< 0.001	18.480	1.814	0.037	7.012
M374T236	1.478	< 0.001	0.036	2.135	0.002	0.012
Uric acid	1.092	0.029	1.963	0.844	0.107	1.270
Urea	1.270	0.001	2.179	0.135	0.804	1.025

*NP *Normal CP diet, *LP *Low-CP diet, *LPAB *Low-CP amino acid balance diet, *LPAI *Low-CP amino acid imbalance diet, *VIP *Variable importance in the projection scores, *FC *Fold change

^1^Volcano plot analysis provides the *P*-value, which is a combination of the fold change and *t*-test analyses

^2^FC= LP relative quantitation/NP relative quantitation and LPAI relative quantitation/LPAB relative quantitation

^3^All data in the table are expressed as relative quantification (intensity)

**Table 12:** Relative quantitation of important shared differential metabolites in four treatments

Item	NP	LP	SEM	QD-value^1^	NP/LP^2^	LPAB	LPAI	SEM	QD-value^1^	LPAB/LPAI
Oxypurinol^3^	85.757	20.700	13.192	65.057	up	41.469	17.410	5.638	24.059	up
Pantothenic acid	0.326	3.377	0.683	-3.051	down	0.390	7.888	1.624	-7.498	down
D-Octopine	6.117	0.964	0.819	5.153	up	2.919	0.958	0.603	1.961	up
Xanthine	3.017	0.323	0.660	2.695	up	0.758	0.264	0.132	0.494	up
6-Tuliposide B	2.199	0.413	0.310	1.785	up	1.019	0.463	0.170	0.556	up
Uridine 5′-monophosphate (UMP)	0.168	1.375	0.175	-1.207	up	0.641	1.345	0.276	-0.704	up
Thiamine	0.913	0.272	0.211	0.640	down	0.939	0.227	0.128	0.712	down
N-(4-Hydroxyphenyl)glycine	1.113	0.225	0.268	0.888	up	0.671	0.142	0.072	0.529	up
Pyridoxal (Vitamin B6)	1.113	0.225	0.268	0.888	up	0.671	0.142	0.072	0.529	up
5′-Phosphoribosylglycinamide	0.722	0.111	0.239	0.611	up	1.050	0.087	0.327	0.963	up
Gly-His	1.051	0.044	0.255	1.008	up	0.779	0.022	0.190	0.757	up
1-Palmitoyl-2-arachidonoyl-sn-glycero-3-phosphoserine	0.038	0.744	0.099	-0.706	up	0.251	0.824	0.223	-0.573	up
Chenodeoxycholic acid	1.650	0.031	0.459	1.619	up	0.087	0.019	0.032	0.068	up
Deoxycholic acid	1.650	0.031	0.459	1.619	down	0.087	0.019	0.032	0.068	down
Hyodeoxycholic acid	1.650	0.031	0.459	1.619	up	0.087	0.019	0.032	0.068	up
Isodeoxycholic acid	1.650	0.031	0.459	1.619	up	0.087	0.019	0.032	0.068	up
Ursodeoxycholic acid	1.650	0.031	0.459	1.619	up	0.087	0.019	0.032	0.068	up
Arachidonic acid (AA)	1.138	0.128	0.370	1.010	up	0.394	0.114	0.107	0.281	up
4-Amino-4-deoxychorismate	0.922	0.134	0.142	0.788	up	0.384	0.113	0.081	0.270	up
2-Piperidone	0.590	0.175	0.198	0.416	up	0.286	0.119	0.065	0.166	up
D-galacto-Hexodialdose	0.548	0.046	0.094	0.502	up	0.439	0.042	0.114	0.397	up
N-Oleoyl glycine	0.834	0.007	0.293	0.827	up	0.108	0.007	0.025	0.101	up
Linoleoylglycine	0.786	0.004	0.235	0.781	up	0.144	0.006	0.040	0.138	up
1-Stearoyl-2-arachidonoyl-sn-glycero-3-phosphoserine	0.031	0.361	0.050	-0.330	up	0.127	0.373	0.081	-0.246	up
5-Tetradecynoic acid	0.527	0.055	0.116	0.472	up	0.205	0.029	0.061	0.176	up
Hygric acid	0.268	0.050	0.089	0.218	down	0.346	0.032	0.124	0.314	down
Pipecolic acid	0.268	0.050	0.089	0.218	up	0.346	0.032	0.124	0.314	up
2-Oxo-4-phosphonobutanoate	0.322	0.043	0.074	0.279	up	0.133	0.037	0.032	0.096	up
M345T195	0.295	0.005	0.046	0.290	up	0.128	0.003	0.058	0.126	up
Dephospho-CoA	0.007	0.203	0.045	-0.196	up	0.016	0.116	0.029	-0.100	up
M368T281	0.195	0.010	0.044	0.185	down	0.104	0.010	0.032	0.094	down
Pantetheine 4′-phosphate	0.007	0.153	0.035	-0.146	up	0.009	0.124	0.036	-0.116	up
Eglumegad	0.115	0.032	0.023	0.083	down	0.064	0.028	0.011	0.036	down
N-Arachidonylglycine	0.159	0.001	0.051	0.158	up	0.052	0.002	0.015	0.050	up
N-Acetylmannosamine	0.136	0.017	0.027	0.119	up	0.028	0.014	0.005	0.014	up
Pyridoxamine	0.101	0.022	0.018	0.079	down	0.052	0.018	0.011	0.033	down
Goralatide (acetate)	0.081	0.016	0.014	0.064	down	0.062	0.017	0.017	0.046	down
N-Acetylglucosaminylasparagine	0.070	0.004	0.018	0.066	up	0.026	0.002	0.006	0.024	up
3-beta-D-Galactosyl-sn-glycerol	0.004	0.026	0.004	-0.022	up	0.006	0.022	0.005	-0.016	up
M730T215	0.021	< 0.001	0.006	0.021	up	0.001	< 0.001	0.001	0.001	up
Acetyl-CoA	< 0.001	0.005	0.001	-0.005	up	< 0.001	0.005	0.002	-0.005	up
M374T236	< 0.001	0.003	< 0.001	-0.003	up	0.001	0.005	0.002	-0.004	up
Uric acid	5.618	11.027	1.199	5.410	down	5.787	7.349	0.483	1.562	down
Urea	0.004	0.009	0.001	0.005	down	0.009	0.009	0.000	0.000	down

*NP *Normal CP diet, *LP *Low-CP diet, *LPAB *Low-CP amino acid balance diet, *LPAI *Low-CP amino acid imbalance diet, *QD-value* Quantitation D-value, *SEM *Standard error of the mean

^1^QD-value = NP relative quantitation – LP relative quantitation and LPAB relative quantitation – LPAI relative quantitation

^2^The variation tendency of metabolites was specified as NP/LP > 1 means up; NP/LP < 1 means down; LPAB/LPAI > 1 means up; LPAB/LPAI < 1 means down

^3^All data in the table are expressed as relative quantification (intensity)

**Fig. 6:**
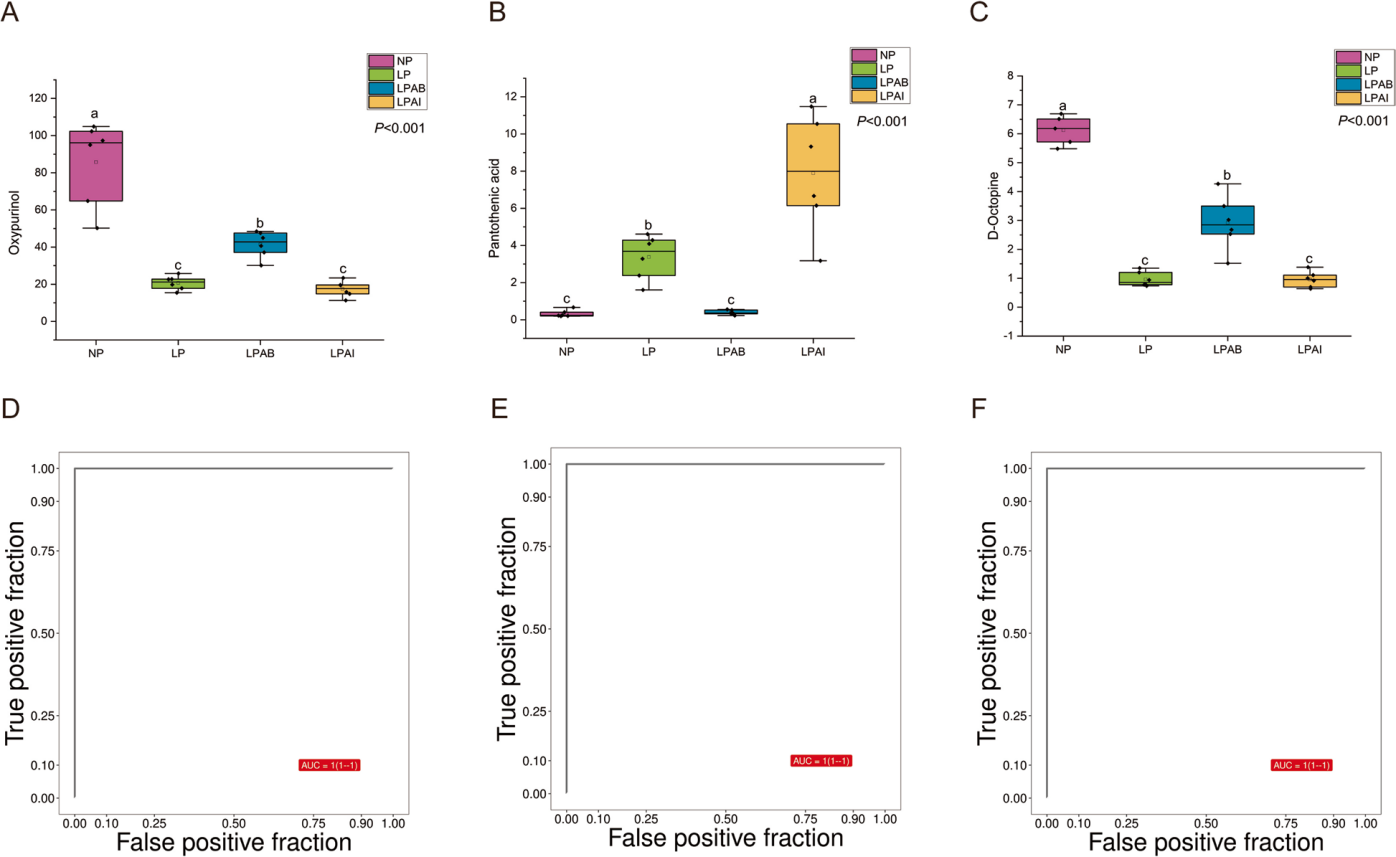
Boxplot of oxypurinol (**A**), pantothenic acid (**B**) and D-octopine (**C**). Receiver operator characteristic (ROC) curve analysis of oxypurinol (**D**), pantothenic acid (**E**) and D-octopine (**F**). The data of 3 metabolites are expressed as relative quantification (intensity)

The potential of metabolites as biomarkers was evaluated using multivariate exploratory ROC curve analysis. Boxplots demonstrated significant differences in these 3 metabolites among the four treatments (Fig. 6A–C), and subsequent ROC analysis revealed that their AUCs were all equal to 1 (Fig. 6D–F), indicating their potential as biomarkers. Consequently, oxypurinol, pantothenic acid, and d-octopine are considered viable candidates for amino acid balance biomarkers.

#### Metabolic pathway analysis

**Fig. 7:**
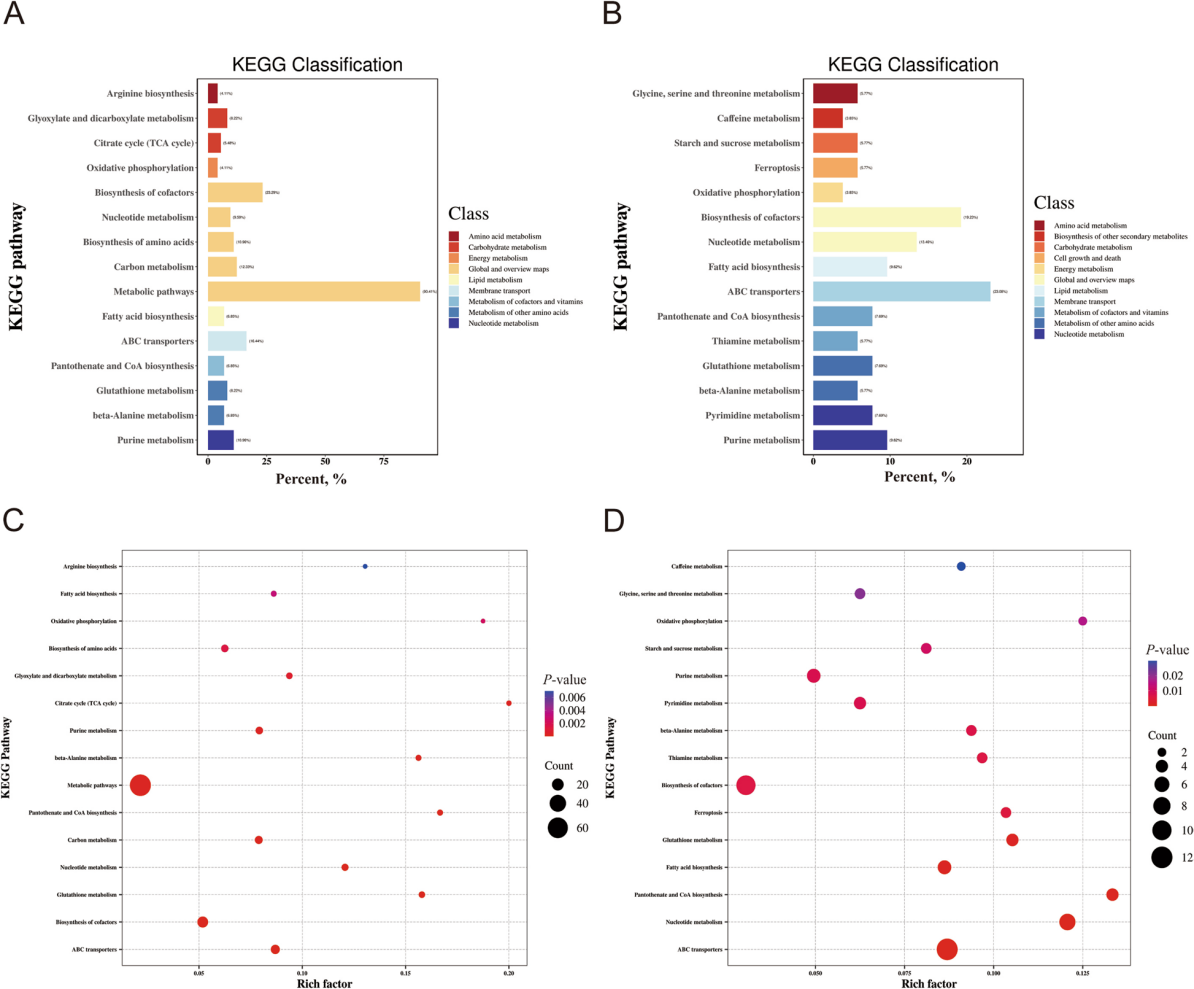
KEGG classification of NP vs. LP (**A**) and LPAB vs. LPAI (**B**). KEGG enrichment bubble of NP vs. LP (**C**) and LPAB vs. LPAI (**D**). NP: Normal CP diet; LP: Low-CP diet; LPAB: Low-CP amino acid balance diet; LPAI: Low-CP amino acid imbalance diet

Pathway analysis revealed the precise impact of amino acid balance alterations on the relevant metabolic networks, and the extent of enrichment in metabolic pathways was quantified using the rich factor (RF) and percentage. Percentages were calculated by dividing the number of differential metabolites in each pathway by the total number of differential metabolites in the sample. RF represents the ratio of differential metabolites to all metabolites within the same pathway (Fig. 7). The significance level was set at *P* < 0.05, with RF > 0.1, and percent > 5. A total of four metabolic pathways were identified: nucleotide metabolism, pantothenate and CoA biosynthesis, fatty acid biosynthesis, and glutathione metabolism (Table 13). These pathways are associated with amino acid balance and can serve as potential targets for future studies on amino acid balance.

**Table 13:** Evaluation score of important shared differential metabolic pathways in four treatments

Metabolic pathways	NP vs. LP			LPAB vs. LPAI		
	Percent, %^1^	RF^2^	*P*-value	Percent, %	RF	*P*-value
ABC transporters	4.110	0.188	0.002	3.846	0.125	0.016
Nucleotide metabolism	6.849	0.167	< 0.001	7.692	0.133	< 0.001
Pantothenate and CoA biosynthesis	8.219	0.158	< 0.001	7.692	0.105	0.001
Fatty acid biosynthesis	6.849	0.156	< 0.001	5.769	0.094	0.007
Glutathione metabolism	9.589	0.121	< 0.001	13.462	0.121	< 0.001
Biosynthesis of cofactors	4.110	0.130	0.007	3.846	0.087	0.032
Thiamine metabolism	4.110	0.097	0.016	5.769	0.097	0.006
beta-Alanine metabolism	16.438	0.087	< 0.001	23.077	0.087	< 0.001
Pyrimidine metabolism	6.849	0.086	0.003	9.615	0.086	0.001
Purine metabolism	4.110	0.081	0.026	5.769	0.081	0.010
Starch and sucrose metabolism	5.479	0.083	0.009	5.769	0.063	0.021
Oxidative phosphorylation	8.219	0.094	0.001	5.769	0.047	0.044
Glycine, serine and threonine metabolism	10.959	0.079	< 0.001	9.615	0.050	0.008
Metabolic pathways	5.479	0.063	0.024	7.692	0.063	0.008
Lysine degradation	5.479	0.071	0.016	5.769	0.054	0.031
Arginine biosynthesis	23.288	0.052	< 0.001	19.231	0.030	0.006
Glyoxylate and dicarboxylate metabolism	90.411	0.022	< 0.001	84.615	0.014	0.031

*NP *Normal CP diet, *LP *Low-CP diet, *LPAB *Low-CP amino acid balance diet, *LPAI *Low-CP amino acid imbalance diet, *RF *Rich factor

^1^ Percent= Percent of the number of differential metabolites in a pathway to the number of all differential metabolites in the sample

^2^RF= The ratio of the number of differential metabolites to the number of all metabolites in the same pathway

## Discussion

After adjusting for the patterns of EAA combinations in low-protein diets, significant differences were observed in various broiler performance indices, indicating the effectiveness of improving animal performance through EAA pattern adjustments. Notably, regardless of sex, broilers fed the low-protein control diet exhibited significantly poorer growth performance than those fed the normal-protein control diet. Surprisingly, a slight advantage was observed in broilers of the low-protein control group during the starter phase. This could be attributed to the presence of a yolk sac in chicks and the relatively less vigorous requirement of exogenous nutrients during the initial growth phase [29]. The high protein content in the normal-protein control group provided adequate protein nutrition with minimal feed intake; however, it also resulted in lower energy intake, which negatively affected growth performance during this phase. Once fully absorbed, a rapid improvement in growth performance was evident for broilers in the normal protein control group during the subsequent phases. In terms of carcass characteristics, broilers fed the normal-protein control diet displayed higher breast and fat pad rates, suggesting a balanced amino acid profile in which sufficient amino acids were utilized for protein synthesis rather than oxidative decomposition, leading to fat accumulation within their bodies. Although not significantly different from the normal-protein control group, broilers in the low-protein control group exhibited a reduced breast rate but comparable fat pad rates. These results indicate that although there remained a balance in amino acid ratios within the low-protein control group, insufficient amounts of amino acids were available for optimal protein synthesis, possibly because of the lower total nitrogen content [15].

The growth performance and breast rate of broilers fed the Lysine and Arginine supplemented diets were significantly improved in diets with adjusted lysine and arginine combination ratios for both male and female broilers. Notably, there was a decrease in dressing, half-evisceration, and evisceration rates after reducing the dietary concentrations of lysine and arginine, which was particularly significant in males. Lysine is the second limiting amino acid in broilers and plays an important role in protein synthesis without being involved in various complex physiological functions [30]. After supplementing low-protein diets with lysine, in addition to meeting the requirements for protein synthesis, along with other amino acids, excess lysine is oxidatively decomposed in the body to form substrates for NEAAs synthesis [31]. This leads to increased concentrations of available amino acids and enhanced protein synthesis within the body. The remaining substances that are not involved in synthesis enter the TCA cycle and eventually contribute to fat deposition, thereby explaining why the breast cancer rate increases simultaneously with fat retention when supplemented with lysine and arginine. When the dietary lysine concentration is low, priority is given to allocating amino acids to synthesize proteins essential for the functioning of vital organs and feather development [32]. Consequently, this results in decreased overall meat yield from broilers, that is, reduced dressing, half-eviscerated, and eviscerated rates. Therefore, increasing lysine and arginine levels in low-protein diets is recommended.

The reduction in methionine and cysteine concentrations in the diet resulted in an improvement in FCR in both male and female subjects and a numerical increase in BW. However, it also led to a significant increase in the fat pad rate, whereas the breast rate remained lower than that in the normal protein control group. The role of methionine in poultry has been extensively studied [33, 34]. Apart from its involvement in protein synthesis, methionine can also enhance the overall body metabolism. When the dietary methionine supply is reduced, animals increase their feed intake to meet normal growth demands, thereby improving amino acid availability within the body and subsequently reducing the FCR while increasing the breast rate. Methionine also plays a regulatory role in the lipid metabolism. Insufficient methionine affects CPT1-mediated fatty acid entry into the mitochondria, leading to excessive fat deposition [35]. Conversely, excessive methionine levels can result in hyperhomocysteinemia in broilers, impairing lipid metabolic processes [36]. This explains why a significant increase in the fat pad rate was observed regardless of whether the methionine concentration was upregulated or downregulated in broilers. Therefore, when formulating low-protein diets, large variations in methionine and cysteine concentrations should be avoided.

The growth performance and carcass characteristics of both male and female animals fed diets with adjusted ratios of BCAA combinations were significantly inferior to those of the control group. BCAAs exhibit antagonistic effects on each other [37]; this study found that adjusting the dietary concentration based on the original BCAA ratio did not prevent the occurrence of these antagonistic effects. This suggests that the interactions between the BCAAs are likely influenced by their absolute concentrations. When adjusting the ratio of BCAA combinations, it is important to consider the weighting of the individual members within the combination. Furthermore, increasing amino acid concentrations according to the current balance patterns when exploring amino acid balance patterns in low-protein diets is not recommended. Additionally, broilers fed a diet containing higher concentrations of BCAAs (the BCAA supplementation group) exhibited worse performance than those fed lower concentrations (the BCAA restriction group), indicating a more pronounced antagonistic effect with increased BCAA concentration. An increase in the grain feed proportion in low-protein diets also leads to an increase in leucine concentration [38]. Leucine is one of the most antagonistic amino acids within BCAAs [39], whereas valine is currently considered the fourth limiting amino acid in poultry [40]. Therefore, future research on BCAAs should consider the relationship between leucine and valine to minimize the negative effects caused by their antagonistic effects.

Growth performance and carcass characteristics were negatively affected by the ratio of dietary threonine after adjustment, with broilers fed a threonine supplemented diet exhibiting the poorest outcomes among all treatments, indicating an extremely unbalanced amino acid pattern. Threonine plays a crucial role in maintaining amino acid balance in the diet and can partially alleviate growth inhibition caused by excessive lysine, methionine, and tryptophan [41]. This suggests that threonine may inhibit the utilization of other amino acids. There have been previous reports on the adverse effects of excess threonine [5]. In future studies, it will be important to meet the minimum requirement of threonine without additional supplementation to maintain an optimal amino acid balance in the diet.

Despite its low dietary concentration, tryptophan cannot be disregarded for its physiological regulatory effects on broilers. Tryptophan supplementation in low-protein diets significantly improved the growth performance and carcass characteristics of both male and female broilers. Notably, the average daily feed intake decreased, which could be attributed to the presence of 5-hydroxy-tryptamine (5-HT), a tryptophan metabolite. 5-HT regulates poultry feeding behavior, and excessive levels can induce satiety and reduce feed intake [42]. Tryptophan stimulates protein synthesis by promoting insulin secretion [43], thereby increasing meat yield. This study revealed that adjusting the tryptophan ratio has a significant effect on thigh yield, particularly in males, while having a minimal effect on breast yield. Further studies are required to better understand these preferences. Moreover, there was no significant increase in the fat pad rate with high concentrations of tryptophan, indicating that supplementing low-protein diets with tryptophan improves the amino acid balance. However, maintenance of animal feed intake without reduction at high tryptophan concentrations should be prioritized in future research.

It is imperative to promptly and accurately assess the dietary amino acid balance to establish a low-protein diet amino acid balance pattern, while elucidating the adjustment trends of each EAA in low-protein diets. Therefore, we first described the entire amino acid metabolism pathway (Fig. 1) and determined different treatments at the transcriptional, protein, and metabolic levels to obtain the optimal and worst amino acid balance treatments for subsequent screening of amino acid balance biomarkers. Based on the results of hepatic enzyme activities and serum metabolites in the amino acid metabolism pathway, it was evident that the threonine supplementation group had the highest levels of both transamination and urinary nitrogen excretion pathways. When there is an imbalance of amino acids in the diet, the amino acids are oxidized and decomposed because of their inability to participate in protein synthesis in a timely manner, leading to increased enzyme activities and metabolite content in the amino acid metabolism pathways [44]. The transamination pathway represents the initiation of amino acid metabolism and decomposition, whereas the gluconeogenesis, fat synthesis, and cholesterol synthesis pathways represent the fate of decomposed amino acids within the body, and the urinary nitrogen excretion pathway indicates the degree of waste from these processes. In the threonine supplementation group, excessive threonine undergoes oxidation and decomposition within the body before being converted into glycine [45], which directly participates in uric acid synthesis [46], resulting in excess nitrogen not being retained as a functional NEAA. This might explain the high nitrogen excretion observed in this treatment. Therefore, from the perspective of protein balance and metabolism, the threonine supplementation group appeared to be the most imbalanced, while the Lysine and Arginine supplementation groups displayed lower enzyme activities and metabolite content across various pathways, indicating greater balance at these two levels. In addition, it was also observed that the tryptophan supplementation group exhibited the highest content in both the fat and cholesterol synthesis pathways. Tryptophan reduces blood glucose levels [47] and promote insulin secretion [48]. Insulin regulates liver metabolism by enhancing fat synthesis, inhibiting fatty acid oxidation, and promoting triglyceride esterification [49]. Consequently, it was anticipated that the tryptophan-supplemented group would exhibit elevated triglyceride and cholesterol levels. Based on the expression of mRNA related to protein synthesis in breast muscle, it was noted that the threonine supplementation group displayed the lowest expression for protein synthesis, while exhibiting the highest expression for degradation. Conversely, this trend was reversed in the lysine- and arginine-supplemented groups, indicating an imbalance at the transcriptional level within the threonine-supplemented group compared with the more balanced state within the lysine- and arginine-supplemented groups.

**Fig. 8:**
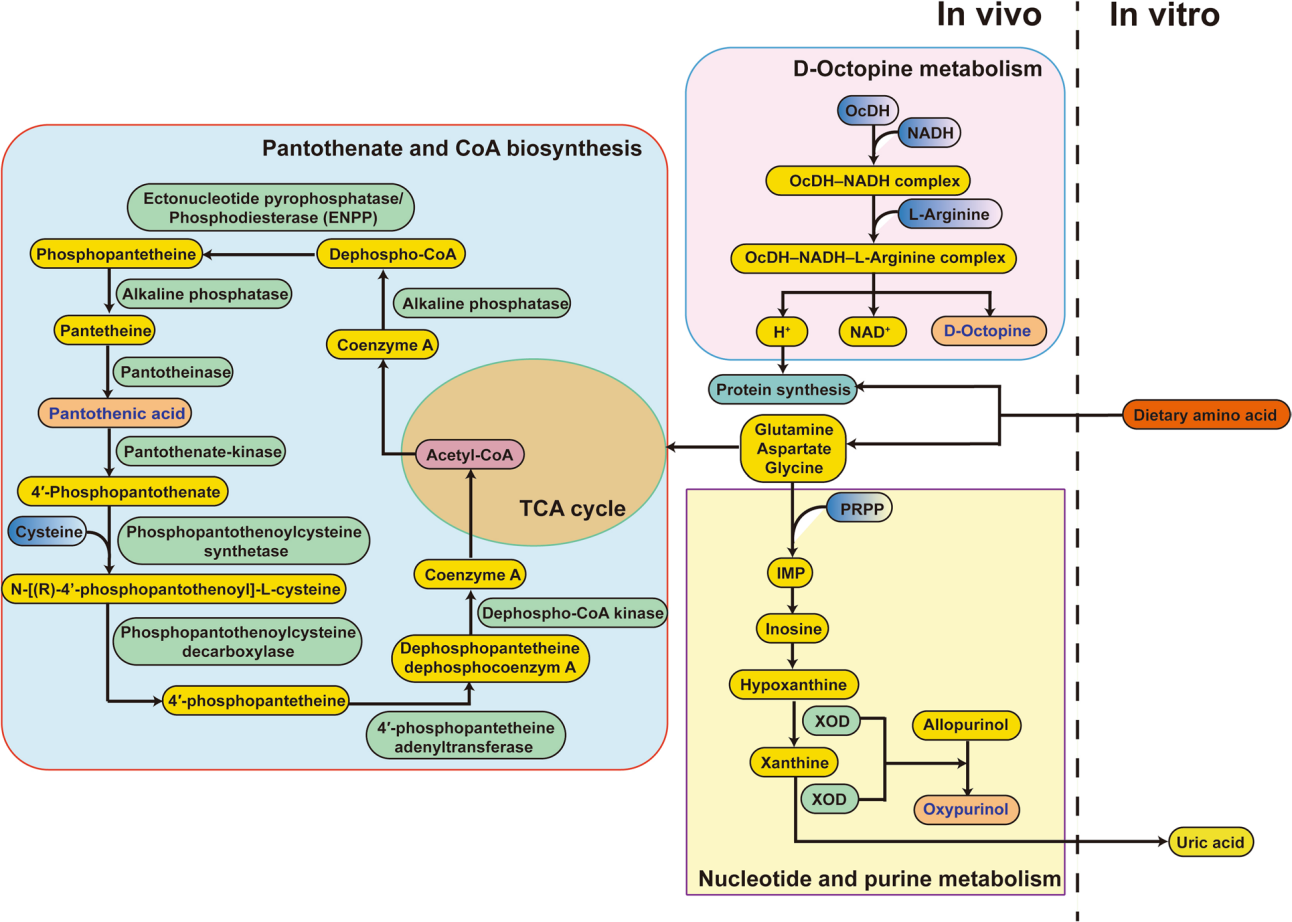
Related pathways of amino acid balance biomarkers involved in amino acid metabolism

Based on the results obtained at the 3 levels, the normal-protein control group was designated as the normal control diet (NP), the low-protein control group as the low-protein control diet (LP), the lysine and arginine supplementation group as the low-protein amino acid balance diet (LPAB), and the threonine supplementation group as the low-protein amino acid imbalance diet (LPAI) for untargeted metabolomic analysis aimed at exploring the biomarkers of amino acid balance. Serum is commonly used to assess the physiological state of an animal. However, serum biochemical indicators are often influenced by animal activities, such as stress, leading to the detection of metabolites unrelated to the experiment and compromising study accuracy when exploring metabolism and identifying biomarkers. Considering that the liver serves as the primary site for amino acid metabolism, metabolites are synthesized and transferred into the bloodstream through the portal vein, and investigating changes in amino acid metabolism in the liver tissue provides a more representative approach. Therefore, liver tissue was selected for analysis in this study. Following metabolite identification and statistical analysis, 3 metabolites were identified as biomarkers of the amino acid balance: Oxypurinol, pantothenic acid, and D-octopine. Their associations with amino acid metabolism are shown in Fig. 8. Oxypurinol is an active metabolite and analog of xanthine formed through the oxidation and decomposition of its precursor, allopurinol [50]. Allopurinol, which resembles hypoxanthine, competes with xanthine oxidase (XOD) during uric acid formation. It is commonly used to treat gout [51]. The primary metabolic mechanism involves the XOD-mediated conversion of allopurinol to oxypurinol by rotation at its enzyme active site, leading to the reduction of the Mo (VI) active center to Mo (IV), thereby inhibiting uric acid production [52]. In this study, oxypurinol content significantly increased in both amino acid balance treatments (NP and LPAB), despite no exogenous supplementation of allopurinol. This can be attributed to only a small amount of surplus amino acids entering catabolic processing under amino acid balance conditions, resulting in insufficient NEAAs such as aspartic acid, glutamine, and glycine, which serve as raw materials for de novo purine synthesis [53]. Consequently, there is a decrease in hypoxanthine and xanthine content, the original substrates for XOD, which enhances the competitiveness of allopurinol, leading to an increased generation of oxypurinol. Additionally, we observed an increase in xanthine concentration (Table 12); this finding is not contradictory. The absolute utilization of XOD by allopurinol inhibits the timely excretion of xanthine, similar to that of uric acid. However, it cannot be converted back to hypoxanthine and thus accumulates in significant quantities in the body. Notably, oxypurinol exhibited the highest relative abundance among all metabolites and displayed substantial variation across different treatments. Importantly, it demonstrates a close association with the end products of amino acid metabolism. Therefore, we propose that oxypurinol is the most representative biomarker for assessing amino acid balance.

Homeostatic regulation of pantothenic acid and vitamin B_5_ in the body involves their interactions with pantothenic acid and coenzyme A [54]. In the presence of pantothenate deficiency, coenzyme A is dephosphorylated by alkaline phosphatase to form dephospho-CoA, which then undergoes hydrolysis of its 5′-phosphodiester bond to yield phosphopantetheine. The subsequent step involves the synthesis of pantetheine, catalyzed by alkaline phosphatase, which is ultimately converted to pantothenic acid [55]. In the absence of coenzyme A, pantothenic acid is phosphorylated to 4′-phosphopantothenate by pantothenate-kinase kinase, which subsequently condenses with cysteine. Decarboxylase removes the carboxyl group from this compound to generate dephosphocoenzyme A, which eventually leads to coenzyme A [56]. The amino acid-balanced diets (NP and LPAB) were expected to exhibit reduced pantothenic acid content in this study. The ultimate goal of amino acid metabolism is to produce coenzyme A, which enters the tricarboxylic acid cycle and participates in other substance synthesis processes [57]. As mentioned earlier, when amino acids are balanced, fewer amino acids participate in catabolic processes, resulting in decreased levels of their decomposition products such as coenzyme A (Table 12). Under homeostatic regulation, synthesis reactions favor conversion towards increased levels of coenzyme A, leading to a decrease in the overall pantothenic acid content.

The energy metabolite d-octopine, derived from the combination of octopine dehydrogenase (OcDH) and NADH [58], undergoes a series of metabolic reactions: first, NADH binds to domain 1 of OcDH to form a complex; subsequently, arginine binds to domain 2 of OcDH, resulting in the formation of an OCDH-NADH-L-arginine complex; and finally, pyruvate binds to the last binding site within the complex and promptly condenses into D-octopine while releasing H^+^ and NAD^+^ [59, 60]. In an amino acid-balanced diet, where there is an abundance of raw materials for protein synthesis, both the quantity and rate of protein synthesis increase with higher energy requirements. Consequently, a significant increase in D-octopine content was observed in the NP and LPAB groups. During amino acid utilization, ensuring a coordinated energy supply is crucial for animal growth [61]. This is particularly true when animals are on an amino acid-balanced diet because rapid protein synthesis may lead to insufficient energy levels, causing non-participating amino acids to enter catabolic processes, thereby affecting the evaluation index values. Such circumstances can result in erroneous assessment of dietary amino acid balance patterns. Therefore, it would be meaningful to conduct experiments on dietary amino acid balance patterns while ensuring an adequate energy supply for animals.

These 3 metabolites are not commonly observed in the investigation of dietary amino acids. Therefore, we selected the common metabolites uric acid and urea from the metabolomic data for comparative analysis (Tables 11 and 12). However, these 2 metabolites exhibited relatively low evaluation scores, and their differences were insignificant when minor variations in the degree of balance of dietary amino acids were considered. In particular, the urea content was within that of the LPAB and LPAI groups. These findings suggest that the previous indicators used to assess amino acid balance lack sufficient sensitivity, especially in studies with minimal differences, which may lead to erroneous evaluations. Oxypurinol is an upstream metabolite of uric acid, whereas pantothenic acid acts upstream of tricarboxylic acid cycle products such as cholesterol and triglycerides. In contrast, D-octopine plays a central role in energy metabolism. They are highly sensitive to the amino acid balance and do not disrupt normal amino acid metabolism. Furthermore, based on the results of metabolic pathway enrichment analysis, oxypurinol corresponded to nucleotide metabolism, whereas pantothenate corresponded to pantothenate and CoA biosynthesis. Therefore, these 3 metabolites serve as reliable indicators for assessing the amino acid balance. In the subsequent amino acid balance study, the evaluation was based on the direction of the metabolite changes provided in Table 12. Specifically, higher levels of oxypurinol and d-octopine, along with lower levels of pantothenic acid, indicated a more balanced composition of dietary amino acids in this group.

## Conclusion

In conclusion, adjusting the patterns of EAAs in low-protein diets is required to enhance amino acid balance and improve broiler growth performance. Untargeted metabolomic analysis identified 43 metabolites and 4 metabolic pathways associated with amino acid balance, including hepatic oxypurinol, pantothenic acid, and D-octopine, as potential biomarkers for assessing dietary amino acid balance. By detecting these biomarkers, the degree of dietary amino acid balance can be precisely evaluated. This study provides valuable insights for further exploration of amino acid balance patterns in low-protein diets of broilers.

## Supplementary Information

Additional file 1: Table S1. Ingredients and nutrient composition of the experimental diets in starter phase (as-fed basis). Table S2. Ingredients and nutrient composition of the experimental diets in grower phase (as-fed basis). Table S3. Ingredients and nutrient composition of the experimental diets in finisher phase (as-fed basis).

Additional file 2: Fig. S1. Effects of amino acid combinations with different patterns on alterations of mRNA expressions in the breast muscle of AA broilers. Fig. S2. Orthogonal partial least-squares discriminant analysis (OPLS-DA) of NP vs. LP liver metabolomics data (A) and LPAB vs. LPAI liver metabolomics data (B). The permutation test (100 times) of the OPLS-DA model of NP vs. LP (C) and LPAB vs. LPAI (D).

## Data Availability

The datasets presented in this paper can be found in online repositories. The names of the repository/repositories and their accession numbers(s) can be found in the article/supplementary material.

## Abbreviations

ACC: Acetyl CoA carboxylase
ADFI: Average daily feed intake
ADG: Average daily gain
ALB: Albumin
ALT: Alanine transaminase
AST: Aspartate aminotransferase
AUC: Area under the curve
BCAA: Branched-chain amino acid
BR: Breast rate
BUN: Blood urea nitrogen
BW: Body weight
DR: Dressing rate
EAA: Essential amino acid
ER: Eviscerated rate
FAS: Fatty acid synthase
FCR: Feed conversion ratio
FR: Fat pad rate
G6PC: Glucose-6-phosphatase
GLB: Globulin
GLU: Glucose
HMGR: HMG CoA Reductase
HR: Half-eviscerated rate
KEGG: Kyoto Encyclopedia of Genes and Genomes
LP: Low-protein diet
LPAB: Low-protein amino acid balanced diet
LPAI: Low-protein amino acid imbalance diet
LPD: Low-protein-level diets
NEAA: Nonessential amino acid
NP: Normal diet
NPD: Normal-protein-level diets
OcDH: Octopine dehydrogenase
OPLS-DA: Orthogonal partial least squares discriminant analysis
PC: Pyruvate carboxylase
PCA: Principal component analysis
ROC: Receiver operating characteristic curve
TC: Total cholesterol
TG: Triglyceride
TP: Total protein
TR: Thigh rate
UA: Uric acid
XOD: Xanthine oxidase
