# Supplementary material for: Assessment of the dietary amino acid profiles and the relative biomarkers for amino acid balance in the low-protein diets for broiler chickens
Source: J Anim Sci Biotechnol. 2024 Nov 14;15:157. doi: 10.1186/s40104-024-01108-2 (PMC11562705; doi:10.1186/s40104-024-01108-2)
Supplement: Supplementary file 2 — Additional file 2: Fig. S1. Effects of amino acid combinations with different patterns on alterations of mRNA expressions in the breast muscle of AA broilers. Fig. S2. Orthogonal partial least-squares discriminant analysis (OPLS-DA) of NP vs. LP liver metabolomics data (A) and LPAB vs. LPAI liver metabolomics data (B). The permutation test (100 times) of the OPLS-DA model of NP vs. LP (C) and LPAB vs. LPAI (D). [file 40104_2024_1108_MOESM2_ESM.docx]

| 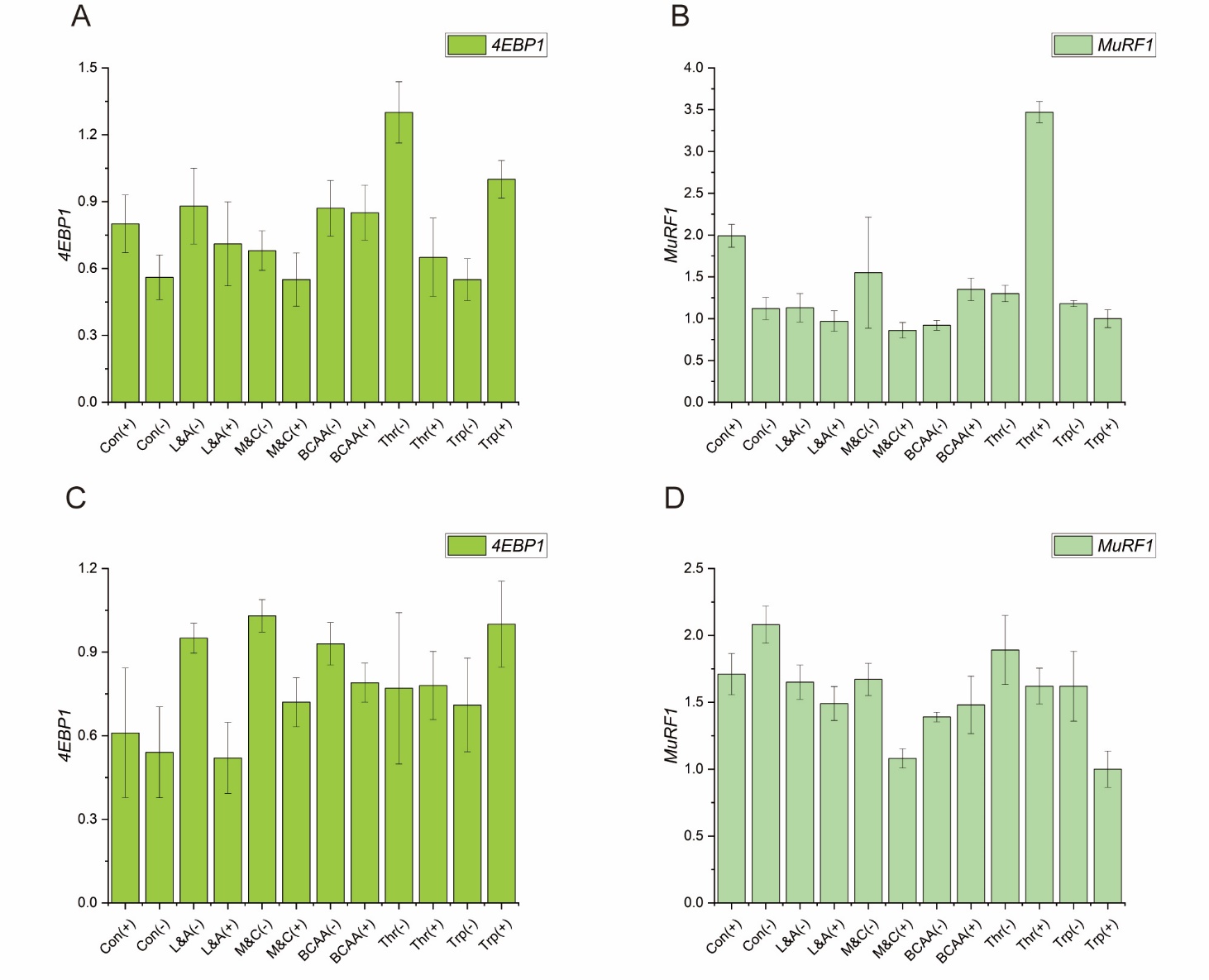 |
| --- |
| **Fig. S1** Effects of amino acid combinations with different patterns on alterations of mRNA expressions in the breast muscle of AA broilers. Con(+): The normal-protein control group; Con(-): The low-protein control group; L&A(-): The lysine and arginine restriction group; L&A(+): The lysine and arginine supplementation group; M&C(-): The methionine and cysteine restriction group; M&C(+): The methionine and cysteine supplementation group; BCAA(-): The BCAA restriction group; BCAA(+): The BCAA supplementation group; Thr(-): The threonine restriction group; Thr(+): The threonine supplementation group; Trp(-): The tryptophan restriction group; Trp(+): The tryptophan supplementation group; Male-*4EBP1* (**A**); Male-*MuRF1* (**B**); Female-*4EBP1* (**C**); Female-*MuRF1* (**D**). *4EBP1*: Eukaryotic translation initiation factor 4E-binding protein-1; *MuRF1*: Muscle ring finger 1. ^a–d^Dissimilar letters represent significant difference among different treatments (*P* < 0.05) |

| 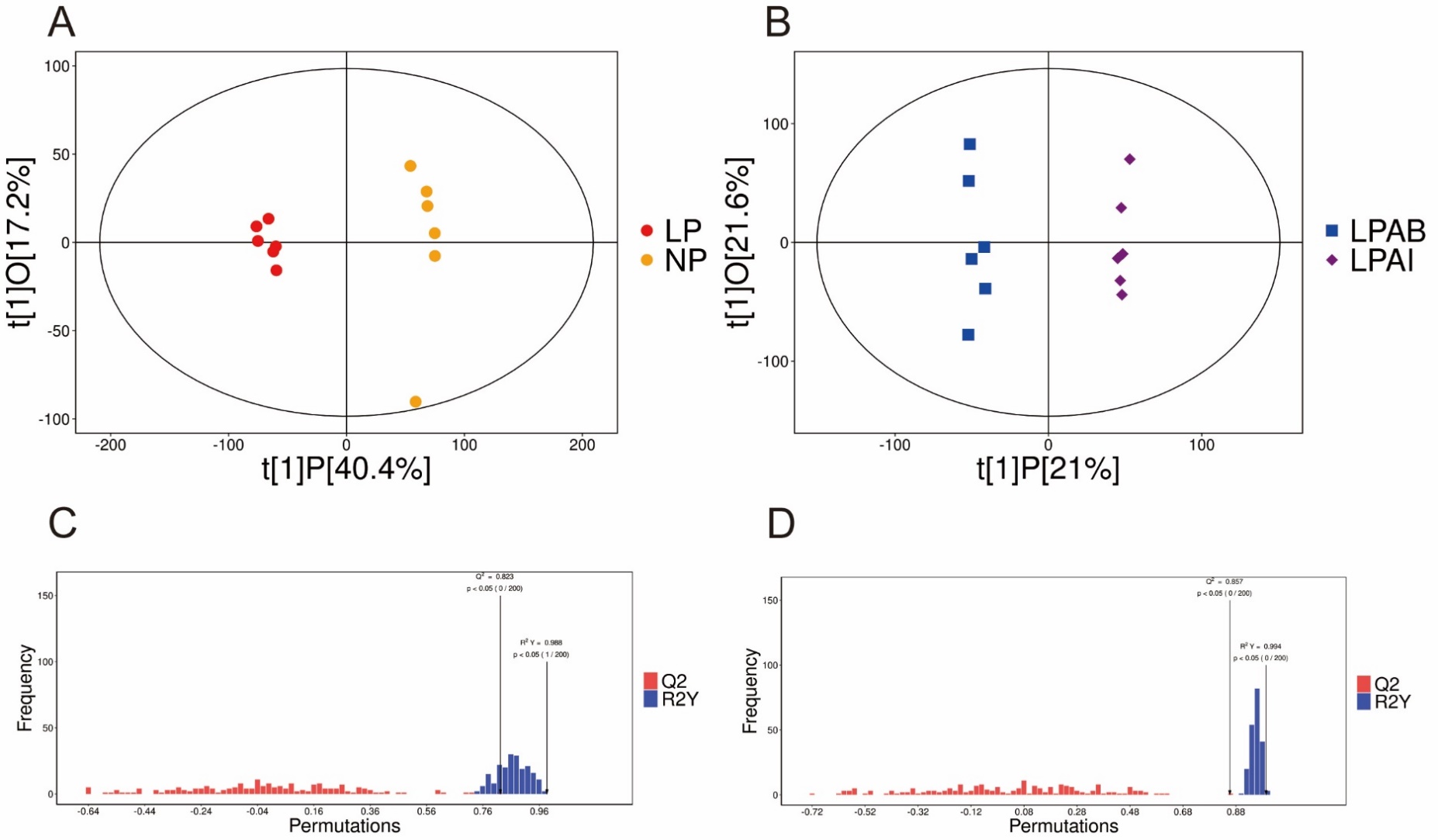 |
| --- |
| **Fig. S2** Orthogonal partial least-squares discriminant analysis (OPLS-DA) of NP vs. LP liver metabolomics data (A) and LPAB vs. LPAI liver metabolomics data (B). The permutation test (100 times) of the OPLS-DA model of NP vs. LP (C) and LPAB vs. LPAI (D). NP: Normal CP diet; LP: Low-CP diet; LPAB: Low-CP amino acid balance diet; LPAI: Low-CP amino acid imbalance diet |
